# Nitrogen-Fixing Bacterium GXGL-4A Promotes the Growth of Cucumber Plant Under Nitrogen Stress by Altering the Rhizosphere Microbial Structure

**DOI:** 10.3390/microorganisms13081824

**Published:** 2025-08-05

**Authors:** Ying-Ying Han, Yu-Qing Bao, Er-Xing Wang, Ya-Ting Zhang, Bao-Lin Liu, Yun-Peng Chen

**Affiliations:** 1 Institute of Biothermal Science and Technology, School of Health Science and Engineering, University of Shanghai for Science and Technology, 516 Jungong Road, Shanghai 200093, China; yyhan2007@163.com (Y.-Y.H.); blliuk@163.com (B.-L.L.); 2 Department of Resources and Environment, School of Agriculture and Biology, Shanghai Jiao Tong University, 800 Dongchuan Road, Shanghai 200240, China; wex.666@sjtu.edu.cn (E.-X.W.); zyting2022@sjtu.edu.cn (Y.-T.Z.); 3 Shanghai Yangtze River Delta Eco-Environmental Change and Management Observation and Research Station (Shanghai Urban Ecosystem Research Station), Ministry of Science and Technology, National Forestry and Grassland Administration, 800 Dongchuan Road, Shanghai 200240, China

**Keywords:** ammonium transporter, soil enzyme, rhizosphere microbe, plant growth promotion, gene deletion mutant

## Abstract

The rhizosphere microbiome plays an important role in carbon- and nitrogen-cycling in soil and in the stress response of plants. It also affects the function of the ammonium transporter (AmtB) that senses nitrogen levels inside and outside the cells of the associative nitrogen-fixing bacterium GXGL-4A. However, the potential mechanism of the interaction between the AmtB deletion mutant of GXGL-4A (∆*amt*B) and microorganisms in the rhizosphere of plants under low-nitrogen stress is still unclear. As revealed by transcriptome analyses, mutation of the *amt*B gene in GXGL-4A resulted in a significant up-regulation of many functional genes associated with nitrogen fixation and transportation at transcription level. The application of ∆*amt*B changed the nitrogen level in the rhizosphere of cucumber seedlings and reshaped the microbial community structure in the rhizosphere, enriching the relative abundance of *Actinobacteriota* and *Gemmatimonadota*. Based on bacterial functional prediction analyses, the metabolic capacities of rhizobacteria were improved after inoculation of cucumber seedlings with the original strain GXGL-4A or the ∆*amt*B mutant, resulting in the enhancement of amino acids, lipids, and carbohydrates in the cucumber rhizosphere, which promoted the growth of cucumber plants under a low-nitrogen stress condition. The results contribute to understanding the biological function of gene *amt*B, revealing the regulatory role of the strain GXGL-4A on cucumber rhizosphere nitrogen metabolism and laying a theoretical foundation for the development of efficient nitrogen-fixing bacterial agents for sustainable agricultural production.

## 1. Introduction

Cucumber is a globally cultivated vegetable, and since 1970, the planting area of cucumber has been continuously increasing due to the establishment of field cultivation facilities. Whether it is protected cultivation or open-air cultivation, excessive use of chemical nitrogen fertilizers in cucumber production can lead to the accumulation of nitrite in cucumbers, thereby affecting the quality of the cucumber. In addition, improper use of nitrogen fertilizers in agricultural ecosystems may lead to environmental problems such as eutrophication of water and soil acidification [1,2]. Therefore, improving the nitrogen utilization efficiency of crops and reducing the environmental pollution caused by chemical nitrogen fertilizers is an urgent problem to be solved.

The completion of cucumber genome sequencing provides a good platform for the construction of large-scale genetic maps and gene cloning of cucumbers. Many studies have shown that adversity stress, including biotic and abiotic factors, can affect the absorption, transport, and assimilation of nitrogen in cucumbers. For example, under low temperature conditions, the absorption of nitrate nitrogen by cucumber roots significantly decreases even with sufficient nitrogen supply. Cucumber is a typical nitrate-preferring plant. When using ammonia nitrogen as the sole nitrogen source, the growth of cucumber seedlings will be inhibited, and, in severe cases, the plants may even wither [3].

Previous research indicated that the application of nitrogen fertilizer can significantly improve the absorption of calcium, magnesium, and zinc in cucumbers, thereby greatly increasing their yield [4]. In recent years, the introduction of low-affinity nitrate transporter genes of cucumber into *Arabidopsis* mutants has successfully restored their nitrate absorption capacity [5]. Zhao et al. analyzed the transcriptome data of cucumber under nitrogen deficiency conditions and found that *MYB12* can regulate auxin and ethylene signaling, thereby promoting the absorption of nitrate by cucumber under nitrogen deficiency conditions [6].

Although the physiological and biochemical responses of plants to different nitrogen nutrient levels have been extensively explored, most research still focus on the forms of nitrogen and the effects of nitrogen nutrient levels on plant growth. Promoting the effect of nitrogen-fixing bacterium (NFB) on plant growth is well confirmed, but the transcriptome modification of NFB, especially the fluctuation pattern of the transcription levels of functional genes involved in nitrogen fixation after inoculation are unclear.

The ammonium transporter AmtB is a channel protein related to the transport of ammonium nitrogen from the extracellular environment and serves as a sensor for extracellular ammonia concentration. The level of AmtB also involves changes in protein membrane localization. Under conditions of external nitrogen limitation, AmtB dynamically exerts transamination on the cytoplasmic membrane in the form of oligomers. Under high ammonium stress, AmtB accumulates and aggregates in the cytoplasm, and its ability to transport ammonium is inhibited [7]. *E. coli* AmtB–GlnK complex plays a physiological function in inactivating the channel. However, the formation of the complex also reduces the cytoplasmic content of GlnK and leads to potential physiological consequences [8]. For every reduction of N_2_, 16 ATP equivalents are used, and Pi is released. Therefore, nitrogen fixation is an energy consuming process, and the expression of nitrogenase is strictly controlled. An efficient way to improve NFB efficiency is through the overexpression of genes with different structures of nitrogenase [9,10].

The membrane protein AmtB-mediated transport of Ammonium (NH_4_^+^) across cell membranes plays an important role in the assimilation or removal of environmental nitrogen. Disruptions in the ammonium transporter gene *amt*B result in the production of sufficient levels of extracellular nitrogen to support the growth of the widely studied model diazotrophic bacterium, *Azotobacter vinelandii* [11]. Recent research reported that AmtB is dispensable for the ammonium uptake in the phototrophic diazotroph *Rhodopseudomonas palustris* harboring two *amt*B genes. The *R*. *palustris* Δ*amt*B1 Δ*amt*B2 mutant, lacking AmtB1 and AmtB2 proteins, was still capable of utilizing NH_4_^+^ regardless of environmental NH_4_^+^ levels. Moreover, the deletion of the *amt*B1 and *amt*B2 genes resulted in the up-regulation of the transcription levels of many transporter genes, providing potential targets for future investigations of alternative NH_4_^+^ uptake systems [12]. These results revealed that the impact of the *amt*B gene on the uptake of NH_4_^+^ from the extracellular environment and maintenance of the stability of the intracellular nitrogen pool varies among diazotrophs. The associative NFB *Kosakonia radicincitans* GXGL-4A had been isolated from maize roots, and it was subjected to a whole genome sequencing. There are a total of 21 core nitrogen fixation-related genes in the genome of GXGL-4A, including 5 nitrogenase structural genes (*nif*s), 5 cofactors (FeMoco), 7 general nitrogen metabolism regulatory genes (1 *ntr*B, 3 *ntr*C, 2 *gln*D and 1 PII), 1 ammonium carrier-encoding gene (*amt*B), 1 nitrogenase regulatory gene (*nif*A), and 2 other *nif* genes [13]. Furthermore, it was found that compared with the wild-type strain GXGL-4A, the *amt*B deletion mutant of *K*. *radicincitans* GXGL-4A (∆*amt*B) had a significantly improved ammonium secretion ability and showed an enhanced growth promoting effect on cucumber plants [14]. However, the promotion mechanism of the NFB strain ∆*amt*B, belonging to the genus of *Kosakonia,* on plant growth is not clear, and no relevant reports have been found. To further unveil the interaction between the mutant and host plants, it is necessary to conduct research on the changes in plant rhizosphere microorganisms after NFB inoculation. The supply level of exogenous nitrogen has a very important influence on the growth and development of cucumber. Insufficient or excessive nitrogen application will lead to metabolic disorders in the body and affect the formation of yield [15]. The associative and endophytic diazotrophic bacterium *Paenibacillus beijingensis* BJ-18 was confirmed to contribute to cucumber seedlings with fixed N, promote plant growth and N uptake, and enhance gene expression and enzyme activities involved in N uptake and assimilation in plants. The inoculation of the NFB strain BJ-18 to the cucumber seedlings enhanced NH_4_^+^–N and NO_3_^−^–N uptake from soil, especially low-nitrogen soil [16]. As a principle model for genetics and genomics research in *Cucurbitaceae*, cucumber (*Cucumis sativus* L.) is extensively cultivated worldwide. In the past decade, scientists have made great progress in a near-complete cucumber reference genome assembly and the development of Cucumber-DB, a multi-omics database (http://www.cucumberdb.com/ (accessed on 18 May 2025)) [17,18]. These endeavors should facilitate advancements in cucumber molecular biology, breeding and the mechanisms underpinning the cucumber–microbe interaction. To gain a deeper understanding of the growth promoting mechanism of NFB on plants and the impact of its application on soil microbiota, cucumber seedlings were inoculated with the *amt*B gene deletion mutant of *K*. *radicincitans* GXGL-4A. A high throughput sequencing of the NFB transcriptome and rhizosphere soil bacterial diversity was carried out to explore the interactions between NFB and soil microbiota.

## 2. Materials and Methods

### 2.1. Bacterial Strains and Cucumber Cultivar

The associative NFB strain *Kosakonia radicincitans* GXGL-4A isolated from maize roots was stored in the China General Microbiological Culture Collection Center (CGMCC) with a deposit number CGMCC No. 12588 [13]. The ammonium transporter (AmtB) deletion mutant (∆*amt*B) of strain GXGL-4A was obtained via homologous recombination (HR) in previous work [14]. The cucumber “XintaiMici” was from the XintaiMici Cucumber Seed Farm in Taian City, Shandong Province of China.

### 2.2. Cucumber Planting and Rhizosphere Soil Sampling

The cucumber seeds were subjected to surface disinfection using 75% alcohol for 30 s, followed by a sterile water rinse. Then, the seeds were treated with 2% sodium hypochlorite solution (*m*/*v*) for 10 min and finally washed with sterile water three times. The treated seeds were placed on a filter paper moistened with sterile water to germinate at 28 °C in a dark incubator [14].

Bacterial cells of the wild-type strain GXGL-4A and the mutant ∆*amt*B were cultured overnight at 37 °C and 180 rpm in LB liquid medium. Subsequently, these cells were inoculated in a 400 mL LB medium at a volume ratio of 1% and incubated overnight at 37 °C and 180 rpm. The bacterial cells were collected by centrifugation, and the sediments were washed twice with sterile water. Finally, the resuspended cells were diluted to an approximate concentration of 1 × 10^8^ cfu/mL.

The cucumber seedlings were planted in alkaline (pH: 8.91) and nitrogen-limiting soil (vermiculite: promix ratio of 1:1) taken from the farm of Shanghai Jiao Tong University. The original soil was yellow clay containing 58.1 mg/kg of nitrate nitrogen (NO_3_^−^–N) and 5.05 mg/kg of ammonium nitrogen (NH_4_^+^–N). The mixed cultivation substrate was nitrogen-limiting for the growth of a cucumber plant that prefers nitrogen. Five cucumber seedlings were planted in a flowerpot, and a total of 18 pots of cucumber seedlings were divided into three groups (the control group, the WT-GXGL-4A-treated group, and the ∆*amt*B treatment group). The bacterial cells of the NFB strains were inoculated in the roots of the cucumber seedlings at the two-leaf-and-one-heart stage. In total, 20 mL of bacterial cells were applied in each pot, and the seedlings were fertilized 5 times (treated once every 4 d). On the 15th day after the last treatment, the seedlings’ biomass-related traits, including plant height, root length, seedling fresh weight, and root fresh weight, were determined. For each experimental group, three biological replicates were set, and the statistical analyses were conducted using the software SPSS (version 21.0) [19]. Meanwhile, the rhizosphere soils of cucumber were sampled on the 15th day (Day15), the 30th day (Day30), and the 45th day (Day45) after NFB treatment and stored in a −80 °C refrigerator for subsequent experiments.

### 2.3. DNA Extraction, 16S rRNA Gene Amplicon Sequencing and Data Processing

To explore the dynamic changes in bacterial diversity and community structure of cucumber rhizosphere soil after a short-term application of the NFB strain, 16S rRNA amplicon sequencing (16S rRNA deep sequencing) was carried out to monitor the diversity and abundance of bacterial communities in the rhizosphere soils sampled from three treatment groups at three time points. The soil DNA extraction kit (Omega Bio TEK, Norcross, GA, USA) was used to isolate high-purity genomic DNAs from cucumber rhizosphere soil according to the manufacturer’s instructions. The final DNA concentration and purity were determined by ND 2000 (Thermo Scientific, Wilmington, NC, USA), and the DNA quality was detected by 1% agarose gel electrophoresis. The primer pairs targeting the V3–V4 hypervariable region of 16S ribosomal RNA gene, i.e., 338F (5′-ACTCCTACGGGAGGCAGAG-3′)/806R (5′-GGACTACHVGGGTWTCTAAT-3′), were applied in PCR amplification [20]. The 20 µL of PCR reaction mixture contained 5× FastPfu buffer (4 µL), 2.5 mM of dNTPs (2 µL), 0.4 µL of FastPfu DNA polymerase (2 U) from TransGen Biotechnology Co., Ltd. (Beijing, China), 5 µM of each primer (0.8 µL) and 10 ng of template DNA. The PCR reaction was run according to the program: denaturation at 95 °C for 3 min, followed by 27 thermal cycles including denaturation at 95 °C for 30 s, renaturation at 55 °C for 30 s, and extension at 72 °C for 45 s, and eventually the PCR products were extended at 72 °C for 10 min [21]. The purified amplicons were collected in equimolar quantities on the Illumina MiSeq platform (Illumina, San Diego, CA, USA) for paired end sequencing (2 × 300). The software Trimmomatic (version 0.36) was used to perform quality filtering on the original FASTQ file. Also, the software UPARSE (version 11) was adopted to obtain highly accurate operational taxonomic units (OTUs) from microbial amplicon reads. The RDP classifier algorithm (version 11.5) was used to perform classification analysis based on the full SILVA 16S rRNA gene reference database (SSU123) with a confidence threshold of 70%.

### 2.4. Rarefaction Analysis

Rarefaction analysis was carried out using the method of random sampling of sequences, and the rarefaction curves were constructed based on the number of extracted sequences and their corresponding species (such as OTUs) or diversity index. OTUs with over 97% similarity were randomly selected and subjected to the calculation of *α*-diversity index by adopting Mothur (version 1.30.2), and finally the R language (version 3.3.1) tool was used to create a curve graph [22].

### 2.5. RNA Extraction and Transcriptome Sequencing

The NFB strains GXGL-4A and ∆*amt*B were inoculated into A15 nitrogen-free medium at a 1% inoculation rate, and incubated on a shaker at 37 °C and 180 rpm for 48 h. The bacterial cells were harvested by centrifugation and the total RNA was extracted using TRIzol^®^ reagent according to the manufacturer’s instructions (Invitrogen Corp., Carlsbad, CA, USA). Genomic DNA was removed using Dnase I (TaKara, Kusatsu, Japan). RNA quality was analyzed by an Agilent 2100 Bioanalyzer (Agilent Technologies, Pale Alto, CA, USA) and the RNA concentration was measured on a NanoDrop ND-2000 (Thermo Fisher Scientific, Waltham, MA, USA). An RNA-seq library was constructed according to the manual of the TruSeq^TM^ RNA sample preparation Kit from Illumina (San Diego, CA, USA) using 2 μg of total RNA. The ribosomal RNA (rRNA) depletion, instead of poly(A) purification, was carried out using Ribo-Zero^TM^ Magnetic Kit (Epicenter, Madison, WI, USA), and then all mRNAs were broken into short fragments (around 200 nt) by adding fragmentation buffer. Double-stranded cDNA was synthesized using a SuperScript^TM^ Double-Stranded cDNA Synthesis Kit (Invitrogen, Waltham, CA, USA) with random hexamer primers (Illumina). The synthesized cDNA was subjected to end-repair, phosphorylation, and ‘A’ base addition according to Illumina’s library construction protocol. Libraries were selected for cDNA target fragments of 200 bp on a 2% Low Range Ultra Agarose followed by a PCR amplification (15 cycles) using Phusion DNA polymerase (NEB). The paired-end RNA-seq library was sequenced by a 2 × 150 bp double terminal sequencing strategy using an Illumina HiSeq X Ten sequencer after quantification by TBS380. The processing of original images to sequences, base-calling, and quality value calculations were performed using the Illumina GA Pipeline (version 1.6, San Diego, CA, USA), in which 150 bp paired-end reads were obtained. The transcriptome samples of GXGL-4A and ∆*amt*B were sequenced and the clean reads were compared to the genome of *K*. *radicincitans* GXGL-4A. The clean data of each sequencing bacterial sample reached over 3.07 Gb and the Q30 base percentage was above 94.76%. The relative expression level of each transcript was calculated using the software Salmon (version 0.8.2). DESeq2 was used to perform the analyses of differentially expressed genes (DEGs) in this study (version 1.24.0) [23].

### 2.6. Determination of Soil Enzyme Activity

To investigate the effects of bacterial strains on the biochemical processes and fertility in the soil of the cucumber rhizosphere, the activities of soil enzymes related to soil carbon and nitrogen cycling including amylase (S-AL), alkaline protease (S-ALPT), chitinase (S-Chitinase), cellulase (S-CL), and soil urease (S-UE) were determined using the soil enzyme test kits from Suzhou Mengxi Biomedical Technology Corporation (Ltd.) (Suzhou City, China) [14].

### 2.7. Evaluation of Siderophore Synthesis Ability of the GXGL-4A and ∆amtB Strains

Approximately 5 µL of the bacterial cells of strain GXGL-4A and the ∆*amt*B deletion mutant were dripped onto the chrome azurol S (CAS)-agar plate. An equal volume of sterile water was added in the control group. Then, these detection plates were cultivated at 25 °C and the diameters of yellow siderophore halos were measured 24, 48, and 72 h later. The differences in the siderophore-producing capabilities were analyzed according to the sizes of siderophore halos [24].

## 3. Results

The expression levels of transcripts vary, and the sequencing depth required for accurate quantification also differs. Low-expressed transcripts require a greater sequencing depth to ensure accurate quantification. A total of 151,707,956 clean reads were obtained from RNA-Seq, with Q20 and Q30 base percentages surpassing 98% and 94%, respectively (Table S1). The mapping ratio of the clean reads to the unigenes ranged from 97.99% to 98.41%. The genome mapped ratio varied between 98.66 and 99.2% (Table S2). Meanwhile, analyses of the transcriptome sequencing saturation and gene coverage distribution were conducted. Saturation curves can evaluate whether transcripts with different expression levels are accurately quantified under different sequencing depth conditions. The saturation test results of RNA-seq showed that most genes with moderate to high expression levels (i.e., genes with expression levels above 3.5) approached saturation when compared with sequencing reads at 40% alignment level, indicating that the overall quality of saturation was high, and the sequencing depth could cover most expressed genes (Figure S1). The results of the sequencing coverage analysis revealed that the reads obtained from sequencing were evenly distributed across the genes, and there were no obvious biased peaks in the sequencing (Figure S2). Transcriptome sequencing data were unbiased and sufficient for further analysis of gene expression differences.

### 3.1. Transcriptome Sequencing Data

**Figure 1:**
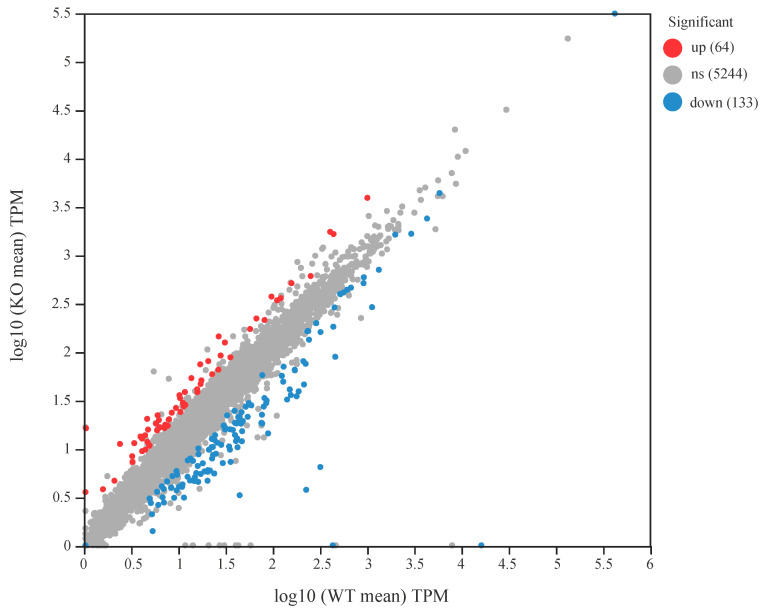
Scatter plot of the gene expression levels between the wild-type strain GXGL-4A and the ∆*amt*B mutant. The symbol ‘ns’ means that there is no significant difference in gene expression at the transcription level between the wild-type strain GXGL-4A and the ∆*amt*B mutant. Up: up-regulated genes at transcription level; down: down-regulated genes at transcription level.

RNA-seq analysis showed that clean data generated for each sample was over 3.07 Gb, and a total of 20.16 Gb of clean data was obtained. Of which 22, 550, 792 to 27, 286, 476 clean reads for each sample were uniquely mapped to the *K. radicincitans* GXGL-4A reference genome sequence, and eventually 5353 expressed genes, including 5167 mRNAs and 186 sRNAs, were detected in this analysis. In total, 197 genes were significantly differentially expressed in the ∆*amt*B mutant; of the DEGs identified, 64 were shown to exhibit significant up-regulation at transcription level; and the expression of 133 functional genes were significantly down-regulated (Figure 1).

### 3.2. Enrichment Analysis Based on the DEGs

Overall, all DEGs were majorly enriched for 20 GO terms, with 14 being independently classified into the biological process (BP), and 4 as the molecular function (MF), and 2 as cell component (CC) (*p* < 0.001). Additionally, many of the DEGs were associated with flagellum assembly and cell motility in the BP category (Figure S3). The down-regulated DEGs in the ∆*amt*B mutant were significantly enriched into 20 GO terms (*p* < 0.001, Figure S4). However, in the up-regulated DEGs, no GO terms were identified at a significant level (*p* < 0.05) (Figure S5). The top 20 KEGG pathways of the identified DEGs included 3 cellular processes (CP), 11 metabolism pathways (M), 1 pathway of environmental information processing (EIP), 3 pathways of human diseases (HD), and 2 organismal systems (OS) (Figure S6). For the down-regulated DEGs in the ∆*amt*B mutant, eight KEGG pathways related to the flagellar assembly; the bacterial chemotaxis and two-component system (CP); the two-component system (EIP); shigellosis, legionellosis, and salmonella infections (HD); the NOD-like receptor signaling pathway; and plant–pathogen interactions (OS) were significantly enriched (*p* < 0.05, Figure S7). For the up-regulated DEGs in the ∆*amt*B mutant, they were significantly enriched into six KEGG pathways including the biosynthesis of siderophore group non-ribosomal peptides, aminobenzoate degradation, atrazine degradation, arginine biosynthesis and C5-branched dibasic acid metabolism (M), and Quorum sensing (CP) (Figure S8).

### 3.3. Rarefaction Analysis of Metagenome Data

Rarefaction curves were made by randomly selecting a certain number of sequences from a sample and calculating the *α*-diversity index of the corresponding samples. In this study, the curves were plotted with the extracted data volume as the horizontal axis and the Sobs and Shannon indices as the vertical axis. The adequacy of the sequencing data volume was assessed via the rarefaction analysis. The results showed that the end of these curves tended to flatten, indicating that the sequencing data of the samples were sufficient and reasonable (Figures S9 and S10). In addition, the OTU coverage curve revealed that the sequencing could represent the real situation of soil microbial diversity accurately (Figure S11). A total of 1, 569, 351 high-quality reads with a mean length of approx. 418 bp and 655, 097, 449 bases were obtained after preprocessing the reads. The results of taxonomic classification using Usearch (version 11) showed a total of 4174 OTUs belonging to 43 phyla, 133 classes, 333 orders, 538 families, 1077 genera, and 2011 species. The top five phyla in terms of microbial abundance were *Proteobacteria*, *Bacteroidota*, *Chloroflexi*, *Actinobacteriota,* and *Firmicutes*.

### 3.4. Soil Enzyme Activity and Seedling Growth Inoculated with NFB Strains Under Low-Nitrogen Stress

**Figure 2:**
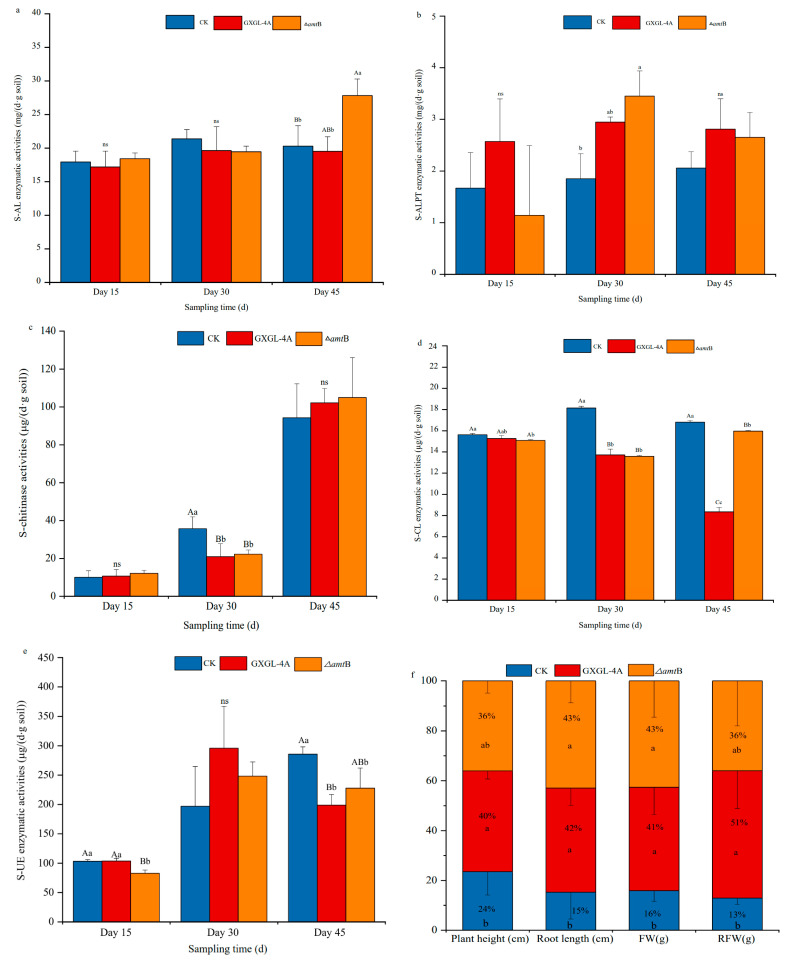
Effects of the different bacterial treatments on soil enzyme activity and cucumber growth under low-nitrogen conditions. (**a**–**e**): The soil enzyme activities of the control, GXGL-4A, and ∆*amt*B treatment groups on Day 15, Day 30, and Day 45 after NFB inoculation. S-AL: soil amylase; S-ALPT: soil alkaline protease; S-CL: soil cellulase; S-UE: soil urease; (**f**): The biomass including cucumber plant height (cm), root length (cm), plant fresh weight (g), and root fresh weight (g) measured on the 15th day after NFB inoculation. Extremely significant differences (*p* < 0.01) and significant differences (*p* < 0.05) are represented by uppercase and lowercase letters, respectively. The data are presented as mean ± SD. The symbol ns means that there is no statistically significant difference between two groups.

The cucumber seedlings were planted in the soils with a low content of nitrogen (pH 8.91, NO_3_^−^–N: 58.1 mg/kg; NH_4_^+^–N: 5.05 mg/kg). The enzyme activities of soil amylase (S-AL), alkaline protease (S-ALPT), chitinase (S-chitinase), cellulase (S-CL), and soil urease (S-UE) were measured after inoculation with the wild-type strain GXGL-4A or ∆*amt*B mutant. On the 15th day, after inoculation on the cucumber seedlings, no significant difference was found in the activity of all the tested soil enzymes between groups (Figure 2a–e). Compared with the control group (i.e., seedlings fertilized with an equal volume of sterile water, designated CK group), the two NFB treatment groups showed similar trends in soil enzyme activity during the experimental period, although the soil enzyme activities varied highly in the ∆*amt*B treatment group.

On the 45th day (day 45) after inoculation, the S-AL activity in the ∆*amt*B treatment group (recorded as KO group) was significantly higher than that in the GXGL-4A treatment group (marked as WT group) (*p* < 0.05), and extremely significantly higher than that in the control group (*p* < 0.01) (Figure 2a). The S-ALPT activity altered in groups on the 30th day (day 30), being significantly higher in the NFB treatment groups than in the control group (*p* < 0.05), and higher in the KO group than in the WT group (Figure 2b). The S-chitinase activities were significantly lower in the NFB treatment groups than that in the control group on day 30, after inoculation (*p* < 0.01) (Figure 2c). There was no significant difference in the S-CL activity between the CK group and the NFB treatment groups on day 15. On day 45, the S-CL activity in the CK group was significantly higher than those in the NFB treatment groups (*p* < 0.01), and similarly the S-CL enzyme activity in the KO group was significantly higher than that of the WT group (*p* < 0.01) (Figure 2d). S-UE activity continuously increased in the CK group. On day 45, the S-UE activity in the CK group was significantly higher than those of the NFB treatment groups (*p* < 0.01). The S-UE activity exhibited the same trend in NFB treatment groups, i.e., first increasing and then decreasing during the whole experimental period (Figure 2e). Because the cucumber plants grew rapidly, the biomass was determined on the 15th day after NFB inoculation. Compared with the plants in the CK group, the cucumber seedlings in the WT and KO groups had a greater biomass (Figure 2f), and the growth parameters, including seedling height, root length, plant fresh weight, and root fresh weight, had a significant difference, with *p* values less than 0.05. The NFB strain (whether GXGL-4A or the ∆*amt*B mutant), as a bio-stimulant, showed a significant plant growth-promoting effect. However, there was no significant difference in the growth of cucumber seedlings between WT and KO groups.

### 3.5. Bacterial Diversity and Composition in the Rhizosphere Soil After NFB Inoculation

**Figure 3:**
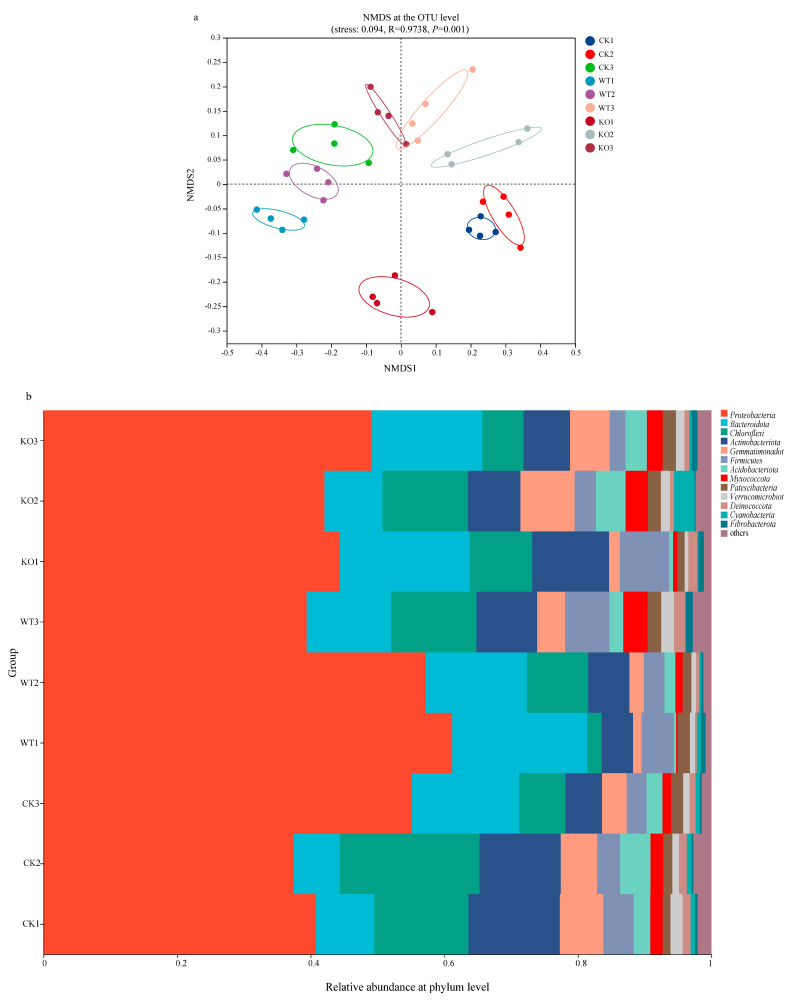

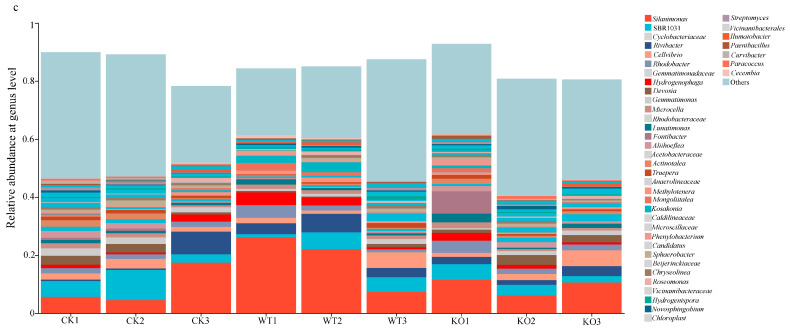
Effects of the low-nitrogen stress on microbial diversity and composition in the cucumber rhizosphere soils treated by the fertilization of NFB bacterial cells. (**a**): Non-metric multidimensional scaling (NMDS) analyses of the rhizosphere soil groups sampled on the 15th, 30th, and 45th days after NFB application (at the OTU level). (**b**): Relative abundance of the dominant bacterial phyla in the rhizosphere soils of cucumber; (**c**): Relative abundance of the dominant bacterial genera in the rhizosphere soils of cucumber. CK: the control group; WT: the GXGL-4A treatment group; KO: the ∆*amt*B treatment group.

The compositions of the bacterial community in the soil were analyzed and the alpha diversity indices between the microbial communities were calculated based on the number of operational taxonomic units (OTUs) in the rhizosphere soil samples (Table 1). After NFB inoculation, the Sobs, Chao 1, ACE, and Shannon indices decreased in the control group (recorded as CK group), suggesting a decrease in the richness and diversity of the bacterial communities. On the contrary, these indices increased in the GXGL-4A treatment group (denoted as WT group), indicating an enhancement in bacterial community richness and diversity. On the 30th day after NFB inoculation, there was no significant difference in bacterial community diversity between the CK group and the NFB treatment groups. On the 45th day since inoculation, the bacterial community richness in the WT group was significantly higher than those of the CK group and the KO group (*p* < 0.01). Compared with the CK group, NFB treatment groups, i.e., the WT and KO groups, showed significant changes in *α*-diversity indices. It should be noted that there was a significant difference in *α*-diversity between the WT and the KO groups (Table 1, *p* < 0.05). The non-metric multidimensional scaling (NMDS) analysis of OTU levels revealed that the bacterial community structure in the rhizosphere soils of cucumber varied significantly after inoculation with the strains GXGL-4A and ∆*amt*B. The bacterial flora in the two NFB treatment groups differed with each other (Figure 3a). In the WT group, the composition of the soil bacterial community on day 45 was significantly different from the microbial profiles on day 15 and day 30. In the KO group, the soil bacterial composition on Day 15 after inoculation was significantly different from those bacterial communities on day 30 and day 45 after the NFB inoculation.

The dominant flora was analyzed at the phylum and genus levels in different experimental groups. The results indicated that the bacterial community structure varied between the experimental groups (Figure 3b). Fourteen phyla were detected in three groups, with the main phyla being *Proteobacteria* (39.8–62.5%), *Bacteroidota* (7.2–21.1%), *Chloroflexi* (2.2–16.7%), and *Actinobacteria* (4.5–13.8%). The four phyla accounted for more than 70% of the total community; thus, they were the dominant phyla in the cucumber rhizosphere soils. On day 15 and day 30 after inoculation with the wild-type strain GXGL-4A and the ∆*amt*B mutant, the proportions of phyla *Proteobacteria* and *Bacteroidota* increased, but the relative abundances of *Chloroflexi* and *Actinobacteriota* decreased. The population proportions of *Proteobacteria* and *Bacteroidota* in the KO group were lower than that of the WT group; however, the relative abundance of the phyla *Chloroflexi* and *Actinobacteria* were high.

The genus *Silanimonas* was the most dominant in the rhizosphere soil microbial communities (4.8–27.2%), and the other microbial taxa including *Cyclobacteriaceae* (0.6–9.5%), *Rivibacter* (0.6–6.7%), and *Cellvibrio* (1.1–5.5%) were identified. The relative abundances of the genus *Silanimonas* and *Cyclobacteriaceae* in the rhizosphere soils differed between the WT and KO groups. Their relative abundances gradually decreased in the WT group but significantly increased in the CK and the KO groups. It is noticed that the relative abundance of the bacterium sbr1031 gradually increased in the WT group (1.09–5.85%) but gradually decreased in the KO group (5.41–2.18%) (Figure 3c). In conclusion, these results indicated that the inoculation of the ∆*amt*B mutant could be reshaping the bacterial community profile in the rhizosphere soil of cucumber.

### 3.6. Microbial Enrichment Analysis and Bio-Functional Prediction in the Cucumber Rhizosphere Soil

**Figure 4:**
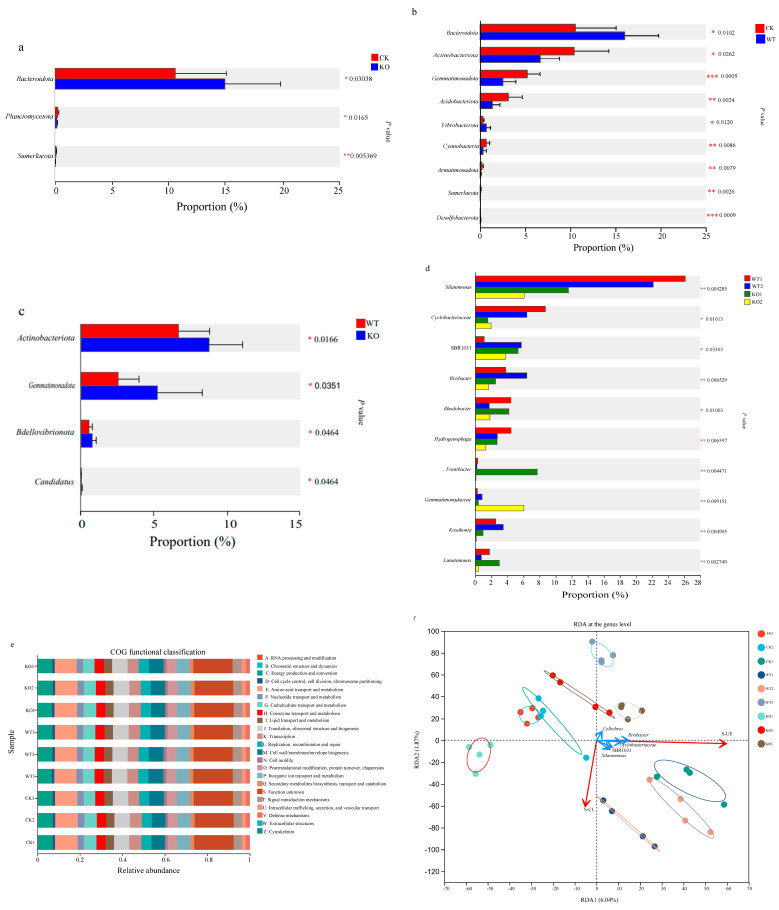
Wilcoxon rank-sum test bar plots at the phylum level. (**a**): CK group vs. KO group. *: *p* < 0.05; **: *p* < 0.01. CK: the control group; KO: the ∆*amt*B treatment group. (**b**): CK group vs. WT group. *: *p* < 0.05; **: *p* < 0.01; ***: *p* < 0.001. CK: the control group; WT: the GXGL-4A treatment group. (**c**): WT group vs. KO group. *: *p* < 0.05. WT: the GXGL-4A treatment group; KO: the ∆*amt*B treatment group. (**d**): The significance difference in the abundance of genus *Kosakonia* between the GXGL-4A- and the ∆*amt*B-treated groups analyzed by the Kruskal–Wallis H test. *: *p* < 0.05; **: *p* < 0.01. (**e**): Functional prediction and COG classifications for the cucumber rhizosphere soil microorganisms based on the COG database using software Picrust2. (**f**): Correlation between the tested soil enzyme activity and microbial community in cucumber rhizosphere soil revealed by using RDA. CK1–CK3, WT1–WT3, and KO1–KO3, respectively, represent the control, the GXGL-4A-, and the ∆*amt*B-treated groups prepared on the 15th, 30th, and 45th day after NFB application. The data are presented as mean ± SD.

There were statistically significant differences in the relative abundances of phyla *Bacteroidota* (*p* < 0.05), *Planctomycetota* (*p* < 0.05), and *Sumerlaeota* (*p* < 0.01) between the KO group and the CK group (Figure 4a). Also, the relative abundances of the phyla *Bacteroidota*, *Actinobacteriota,* and *Fibrobacterota* in the WT group were significantly higher than those in the CK group (*p* < 0.05) (Figure 4b). Similarly, the relative abundances of *Actinobacteriota*, *Gemmatimonadota*, *Bdellovibrionota,* and *Candidatus* in the KO group were significantly higher than those in the WT group (Figure 4c). The Kruskal–Wallis H test was conducted to assess the differences in abundance of the genus *Kosakonia* in the WT and the KO groups. There was an extremely significant difference in the relative abundance of this microbial population between the two groups on the 15th and 30th days after inoculation (*p* < 0.01), but no significant difference was detected on the 45th day after NFB treatment (Figure 4d).

PICRUST2 was applied to predict the pathways associated with the microbial communities. The predicted functions were as follows (Figure 4e): amino acid transport and metabolism (10.29–10.67%), energy production and conversion (6.82–7.27%), translation, ribosomal structure and biogenesis (7.17–7.45%), cell wall/membrane/envelope biogenesis (6.41–7.04%), inorganic ion transport and metabolism (6.04–6.26%), and carbohydrate transport and metabolism (5.39–5.98%). Of the 46 secondary KEGG metabolic pathways enriched in the NFB treatment groups, 12 were related to substance metabolism, including carbohydrate metabolism, amino acid metabolism, energy metabolism, cofactor and vitamin metabolism, and others (Table S1). The relative abundances of 10 secondary KEGG metabolic pathways in the KO group were significantly higher than those in the WT group (*p* < 0.01). These pathways that mainly concerned the metabolisms of amino acids, lipids, nucleotides, and vitamins were up-regulated at transcription level due to the deletion of the *amt*B gene in the genome of strain GXGL-4A.

On the 15th and 30th days after treatment, the dominant bacterial genera in the rhizosphere soils of the KO and the CK groups were *Rhodobacter* and *Hydrogenophaga*, respectively. And, on the 45th day, the dominant genus was *Cellvibrio* in the rhizosphere soil of the WT group (Figure 3c). Redundancy analysis (RDA) at the genus level was used to determine the correlations between the soil enzymes S-UE as well as S-CL and the top five genera in abundance in the NFB-treated rhizosphere soils. The results revealed that the S-CL enzyme activity was positively correlated with the relative abundance of the species in the genus of *Silanimonas* and negatively correlated with the abundance of genera *Cellvibrio*, *Rivibacter,* and *Cyclobacteriaceae*. However, for the S-UE enzyme, its activity was positively correlated with the relative abundance of all the top five genera (Figure 4f). These findings suggest that these dominant bacterial genera in the rhizosphere soils may play an important role in the transformation and utilization of carbon and nitrogen nutrients in microbe–plant interactions.

### 3.7. Transcription Levels of Genes Related to Nitrogen Fixation

**Figure 5:**
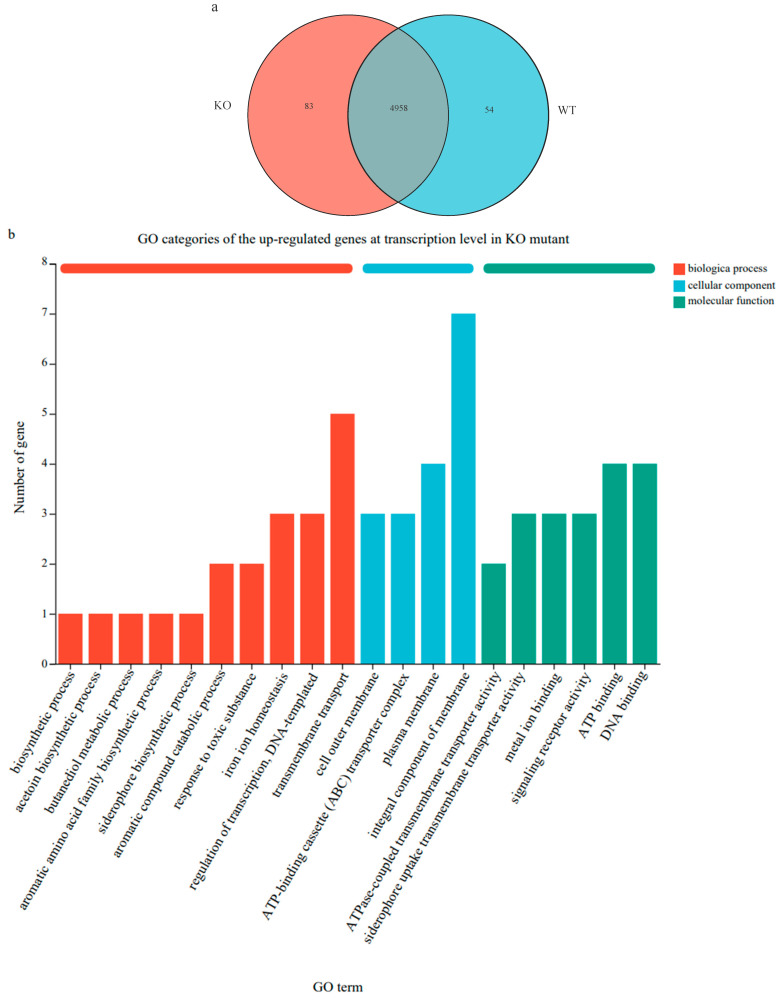

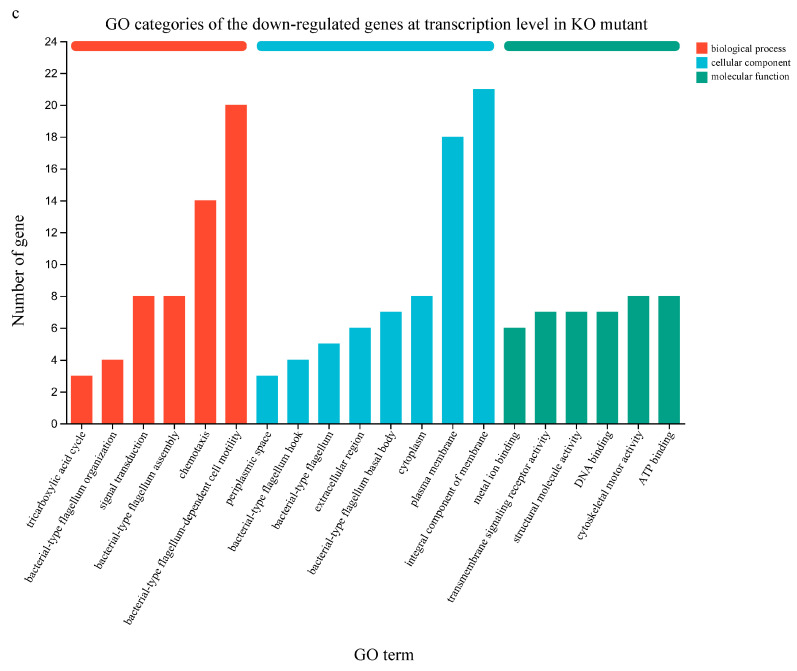
Expression difference in functional genes at transcription level between the wild-type strain GXGL-4A and the ∆*amt*B mutant. (**a**): Venn diagram of gene expression in the bacterial cells of GXGL-4A and its ∆*amt*B mutant. KO: the ∆*amt*B mutant; WT: the wild-type strain GXGL-4A. (**b**): GO categories of the up-regulated genes at transcription level in the ∆*amt*B mutant. (**c**): GO categories of the down-regulated genes at transcription level in the ∆*amt*B mutant.

The differentially expressed genes (DEGs) between the wild-type strain GXGL-4A and the ∆*amt*B mutant were identified based on the screening criteria of the absolute value of the fold change (FC value) > 2 (Figure 5a). A total of 197 DEGs (64 up-regulated and 133 down-regulated genes) were detected. Compared with the wild-type strain GXGL-4A, the ∆*amt*B mutant showed a significant down-regulation in the transcription level of a gene encoding a YJFB family protein (locus tag: A3780_14850).

The transcription level of a functional gene encoding a carrier protein that related to amino acid transport (locus tag: A3780_27015) was significantly up-regulated in the ∆*amt*B mutant. Of the DEGs, 21 nitrogen metabolism-related genes including the *ntr*B gene encoding NRII and the *gln*K gene encoding PII regulatory protein were found. It can be concluded that the transcription levels of nitrogen fixation-related genes were significantly up-regulated in the ∆*amt*B mutant (Table 2).

In the gene ontology (GO) database, genes are classified into categories of process (BP), cellular component (CC), and molecular function (MF) according to the putative function of their encoded products. In this study, the top 20 GO terms enriched with the up-regulated DEGs in the ∆*amt*B mutant are shown in Figure 5b. These GO terms included ‘integral component of membrane’, ‘transmembrane transport’, ‘plasma membrane’, ‘ATP binding’, and ‘DNA binding’. For the GO enrichment of the down-regulated DEGs in the ∆*amt*B mutant, they were mainly enriched in the categories of ‘integral component of membrane’, ‘bacterial-type flagellum-dependent cell motility’, ‘plasma membrane’, and ‘chemotaxis’ (Figure 5c).

## 4. Discussion

This study delineates a hierarchical regulatory network linking bacterial ammonium sensing to rhizosphere microbiome remodeling under nitrogen limitation. By coupling transcriptomic profiling with temporal microbial community analysis, we reveal how AmtB-mediated nitrogen homeostasis in diazotrophs orchestrates plant–microbe metabolic synergy, through both direct genetic regulation and indirect habitat filtering mechanisms. The loss of protein expression of AmtB in the ∆*amt*B mutant triggered a compensatory up-regulation of *nif* (about 3.8 folds) and *ntr* (around 2.7 times) genes at transcription level, mirroring the nitrogen starvation response observed in *Klebsiella oxytoca* M5al [25,26]. Notably, the 4.2-fold induction of *gln*K suggests a conserved PII–AmtB interaction mechanism, where ammonium channel inactivation releases GlnK to activate NtrBC-dependent transcriptional reprogramming [27,28]. This molecular switch may initiate a cascade of rhizosphere events. First, the improvement of nitrogenase activity would increase local NH_4_^+^ leakage and altered root exudate profiles. Secondly, the soil C/N ratio should be modulated through the elevated secretion of pyruvate as a byproduct of the nitrogenase activity. Finally, the redox potential would be shifted via an enhanced H^+^ extrusion during N_2_ fixation [29,30]. The biochemical changes in the soils of the KO group established an ecological filter mechanism favoring the population colonization of *Actinobacteriota* (34.2% (KO) vs. 18.7% (WT)) and *Gemmatimonadota* (12.8% (KO) vs. 5.1% (WT)), whose functional traits synergize with plant needs [31].

The delayed yet sustained increase in S-AL (+142%), S-ALPT (+89%), and S-CL (+116%) activities in the KO groups by Day 45 (Figure 2a,d) correlates with two-phase microbiome restructuring. (1) Phase I (Days 1–30): dominance of the phylum *Proteobacteria* (62.5%) facilitates rapid C mineralization via the genus *Silanimonas* (27.2%). Transient chitinase suppression (−40%) is likely to indicate fungal biomass depletion as alternative N source. (2) Phase II (Days 30–45): enrichment of the phylum *Actinobacteriota* (34.2%) coincides with the 25% increase in polyketide synthase genes (KEGG M00728) and the 18% elevation in ACC deaminase activity. The proliferation of phylum *Gemmatimonadota* aligns with a 32% up-regulation of cytochrome c oxidases (COG1842) at transcription level and an enhanced PHA storage capability (+25% PhaC). This succession pattern mirrors the “microbial service rotation” hypothesis [32,33], where early colonizers prime substrates for late-stage specialists that stabilize plant growth benefits.

The observed 23% biomass increase in the *Δamt*B-treated cucumbers (Figure 2f) may be attributed to several aspects: (1) at the genetic level: the loss of AmtB protein expression can derepress nitrogenase synthesis while up-regulating amino acid transporters (*rs*27015, 4.1 times); (2) at the microbiome level: the microbes in phylum *Actinobacteriota* would enhance the micronutrient bioavailability via siderophores. (3) at the ecosystem level: S-UE/S-CL coupling (RDA, Figure 4f) maintains NH_4_^+^/NO_3_^−^ balance (r = 0.82, *p* < 0.01). Moreover, the increase in S-CL activity leading to an increase in carbon source supply theoretically can enhance the nitrogenase activity of *K*. *radicincitans* GXGL-4A; similar conclusions have been reported in the diazotroph *Klebsiella oxytoca* NG13 [34]. The increase in nitrogenase activity has different effects on the growth of different NFB strains. Scientists took advantage of the aerobic *Azotobacter vinelandii* DJ and an ammonium excreting mutant, AV3 (Δ*Nif*L), to investigate central carbon metabolism fixes and central cell bioenergetics in response to ammonium availability and nitrogenase activity. They found that the respiratory TCA cycle is up-regulated in association with increased nitrogenase activity and causes a monotonic decrease in specific growth rates [35]. However, our previous study showed that the deletion of *amt*B gene had no influence on the growth of bacterial cells [14]. The reason for this difference in growth rate may be that NFB strain GXGL-4A is a facultative anaerobic bacterium, and its cell growth is more adaptable to oxygen supply conditions.

The diversity and abundance of rhizosphere soil microbiota were significantly different between the KO group and the WT group at the early stage after NFB inoculation (Day 15, *p* < 0.05), but there was no significant difference in the abundance of bacterial species between the two groups at the middle and late stages after inoculation (Day 30 and Day 45, *p* > 0.05). The abundance of microbial communities showed a significant difference during the entire experimental period (*p* < 0.05). This result indicates that the application of NFB can rapidly reshape the profiles of soil microbial communities. The application of NFB strains with different ammonium secretion abilities results in a significant difference in the diversity of microbial communities in the rhizosphere soils of cucumber. The abundance of soil microbiota was significantly altered at the early stage (Day 15) after NFB treatment, but there was no significant difference between the two NFB treatment groups at the later stages (Day 30, Day 45) of the experiment, indicating that bacterial diversity is more sensitive to environmental changes compared with the abundance of bacterial communities. The activity of S-UE has a significant impact on the structure and composition of rhizosphere soil bacterial communities; of the top 50 bacterial genera in soil, a total of 22 genera of bacteria shows a significant correlation with S-UE enzyme activity (*p* < 0.05). In addition, a significant correlation was found between the activity of S-CL and the diversity and abundance of 8 dominant bacterial genera in the top 50 genera of the tested rhizosphere soil microbial communities (*p* < 0.05). It should be noted that the bacterial community of the genus *Kosakonia* was significantly affected by S-CL activity, and there was a highly significant negative correlation between the two factors (*p* < 0.001). Overall, the activity of S-ALPT has no significant effect on the profile of soil microbial communities (*p* > 0.05, Figure S12).

Soil biological nitrogen fixation (BNF) plays a significant role in N input in terrestrial ecosystems, and soil NH_4_^+^–N content is the most efficient predictor of BNF rate in a semiarid natural steppe grassland. Soil BNF is believed to be closely associated with soil NH_4_^+^–N content [36]. Other researchers found that soil NH_4_^+^–N concentration, rather than pH or NO_3_^−^–N concentration, was a key environmental parameter determining the composition of the soil bacterial community. NH_4_^+^–N content was thought to be a dominant predictor of bacterial community composition in an acidic forest soil with exogenous nitrogen enrichment [37]. In this investigation, despite growing in the soil with a low NH_4_^+^–N content (5.05 mg/kg, low NH_4_^+^–N stress), the cucumber seedlings showed a good biomass due to the application of NFB strains. The low NH_4_^+^–N soil environment is theoretically more favorable for the ∆*amt*B bacterial cells applied to the soil to exert their ammonium secretion function, thereby achieving a more effective reshaping of the rhizosphere soil bacterial communities.

## 5. Conclusions

This work establishes AmtB as a pivotal node connecting bacterial nitrogen sensing to ecosystem-level microbiome functions. The deletion of the *amt*B gene significantly enhances the transcription levels of *nif* genes in *K. radicincitans* GXGL-4A. After inoculation, the strain ∆*amt*B promotes cucumber plant growth under low-nitrogen stress by regulating the activity of soil enzymes closely related to carbon and nitrogen metabolism in the rhizosphere soils of cucumber and rapidly reshaping the structure of rhizosphere soil microbiota. The results should advance our capacity to design precision biofertilizers for sustainable agriculture in nitrogen-challenged soils.

## Figures and Tables

**Table 1:** Bacterial *α*-diversity analysis at the OTU level in cucumber rhizosphere soil.

**Treatments**	**Sobs**	**Chao 1 Index**	**Shannon Index**	**Ace Index**
Day 15				
CK1	348.67 ± 14.64 Aa	388.06 ± 33.83 Aa	4.43 ± 0.02 Aa	385.93 ± 26.55 Aa
WT1	266.33 ± 12.42 Bc	320.59 ± 11.33 Bb	3.10 ± 0.17 Cc	313.84 ± 6.24 Bb
KO1	318.00 ± 14.00 Ab	364.34 ± 7.98 Aba	3.83 ± 0.15 Bb	364.69 ± 15.25 Aba
Day 30				
CK2	345.00 ± 5.00 Aa	388.97 ± 4.64 Aab	4.17 ± 0.47 Aab	386.39 ± 3.10 Aab
WT2	330.00 ± 14.00 Aa	376.12 ± 15.30 Ab	3.54 ± 0.30 Ab	370.74 ± 14.79 Ab
KO2	342.00 ± 5.00 Aa	415.43 ± 17.69 Aa	4.34 ± 0.09 Aa	394.26 ± 5.71 Aa
Day 45				
CK3	309.33 ± 6.11 Bb	351.18 ± 22.05 Aa	3.81 ± 0.15 Bb	343.00 ± 12.63 Bb
WT3	360.33 ± 14.84 Aa	397.66 ± 26.48 Aa	4.37 ± 0.01 Aa	400.10 ± 25.83 Aa
KO3	304.00 ± 5.57 Bb	349.35 ± 21.75 Aa	4.02 ± 0.11 Bb	345.14 ± 9.64 Bb

Note: CK1–CK3, WT1–WT3, and KO1–KO3, respectively, represent the soil samples of the control group, the GXGL-4A, and the ∆*amt*B treatment groups that prepared on Day 15, Day 30, and Day 45 after NFB fertilization. Sobs, Chao1, Shannon, and Ace indices at the OTU level were obtained. The lowercase letters indicate that there is a significant difference in *α*-diversity index between two groups by *t*-test (*p* < 0.05). The capital letters show a statistically extremely significant difference in *α*-diversity index between two groups by *t*-test (*p* < 0.01).

**Table 2:** The differentially expressed genes (DEGs) in the ∆*amt*B bacterial cells.

**Gene Name**	**Gene ID**	**Gene Description**	**KO**	**WT**
*nif*A	A3780_RS10925	Nif-specific transcriptional activator NifA	398.47	255.33
*nif*B	A3780_RS10920	nitrogenase cofactor biosynthesis protein NifB	145.63	75.25
*nif*D	A3780_RS11000	nitrogenase molybdenum-iron protein alpha chain	177.26	82.29
*nif*E	A3780_RS10980	nitrogenase iron-molybdenum cofactor biosynthesis protein NifE	156.61	78.79
*nif*H	A3780_RS11005	nitrogenase iron protein	478.13	220.91
*nif*J	A3780_RS11010	pyruvate: ferredoxin (flavodoxin) oxidoreductase	167.07	80.1
*nif*K	A3780_RS10995	nitrogenase molybdenum-iron protein subunit beta	172.53	82.61
*nif*L	A3780_RS10930	nitrogen fixation negative regulator NifL	655.41	421.94
*nif*M	A3780_RS10940	nitrogen fixation protein NifM	57.12	27.28
*nif*N	A3780_RS10975	nitrogenase iron-molybdenum cofactor biosynthesis protein NifN	94.81	47.1
*nif*Q	A3780_RS10915	nitrogen fixation protein NifQ	65.56	33.61
*nif*S	A3780_RS10960	cysteine desulfurase NifS	317.63	168.14
*nif*T	A3780_RS10990	putative nitrogen fixation protein NifT	269.04	108.4
*nif*U	A3780_RS10965	Fe-S cluster assembly protein NifU	404.88	215.52
*nif*W	A3780_RS10950	nitrogen fixation protein NifW	95.42	42.73
*nif*B/*nif*X	A3780_RS10970	NifB/NifX family molybdenum-iron cluster-binding protein	37.47	18.93
*nif*B/*nif*X	A3780_RS10985	NifB/NifX family molybdenum-iron cluster-binding protein	182.18	84.06
*nif*Z	A3780_RS10945	nitrogen fixation protein NifZ	81.27	40.4
*anf*D	A3780_RS13465	nitrogenase iron-iron protein alpha chain	3.8	2.02
*anf*G	A3780_RS13460	Fe-only nitrogenase subunit delta	5.55	4.83
*anf*K	A3780_RS13455	Fe-only nitrogenase subunit beta	5.64	3.16
*ntr*B	A3780_RS19230	nitrate ABC transporter permease	89.38	48.8
*amt*B	A3780_RS04740	ammonium transporter AmtB	0	413.75
*gln*K	A3780_RS04735	P-II family nitrogen regulator	1636.51	421.79

## Data Availability

The genomic sequence of the *K. radicincitans* GXGL-4A has been deposited in GenBank database (accession number CP015113). The 16S ribosomal RNA gene amplicon sequencing data have been stored with the SRA Data Libraries under Accession No. SUB14181175 and BioProject No. PRJNA1070836. Transcriptome sequencing data are available in SRA Database (Accession No. SUB15310283, BioProject No. PRJNA1261591).

## Supplementary Materials

The following supporting information can be downloaded at: https://www.mdpi.com/article/10.3390/microorganisms13081824/s1, Figure S1: Saturation curves of RNA-seq. The sequencing saturation analysis of transcriptome data was conducted in the wild-type strain GXGL-4A and its Δ*amt*B mutant. The horizontal axis represents the percentage of valid alignment reads (e.g., 60 represents randomly selecting 60% of alignment sequencing data to calculate the expression level of the gene). The vertical axis represents the deviation ratio between the expression level and the final value under the sampling conditions (within 15% error). Figure S2: Coverage curves of transcriptome data. The horizontal axis represents the percentage of base length of a single gene to the total base length, with 0 indicating the 5′ end of the gene and 100 referring the 3′ end of the gene. The vertical axis shows the total number of sequences within the corresponding interval compared to the horizontal axis positions of all genes. KO: the Δ*amt*B mutant. WT: the wild-type stain GXGL-4A. Figure S3: GO enrichment analysis of the DEGs between the wild-type strain GXGL-4A and the Δ*amt*B mutant. The symbol ‘***’ means that there is an extremely significant enrichment in this GO term (*p* < 0.001). Figure S4: GO enrichment analysis of the down-regulated DEGs between the wild-type strain GXGL-4A and the Δ*amt*B mutant. ***: *p* < 0.001. Figure S5: GO enrichment analysis of the up-regulated DEGs between the wild-type strain GXGL-4A and the Δ*amt*B mutant. Figure S6: KEGG enrichment analysis of the identified DEGs between the wild-type strain GXGL-4A and the Δ*amt*B mutant. The symbol ‘***’ exhibits that there is an extremely significant enrichment in this KEGG pathway (*p*<0.001). Figure S7: KEGG enrichment analysis of the down-regulated DEGs between the strain GXGL-4A and the Δ*amt*B mutant. *: *p* < 0.05; **: *p* < 0.01; ***: *p* < 0.001. Figure S8: KEGG enrichment analysis of the up-regulated DEGs between the strain GXGL-4A and the Δ*amt*B mutant. *: *p* < 0.05; **: *p* < 0.01. Figure S9: Rarefaction curves of the bacterial 16S rRNA gene amplicon paired-end sequencing data (Sobs index at the OUT level). The rhizosphere soils were sampled from the control (CK) group, and the GXGL-4A (WT) and the Δ*amt*B (KO) treatment groups, respectively. Figure S10: Rarefaction curves of the bacterial 16S rRNA gene amplicon paired-end sequencing data (Shannon index at the OUT level). The rhizosphere soils were sampled from the control (CK) group, and the GXGL-4A (WT) and the Δ*amt*B (KO) treatment groups, respectively. Figure S11: Coverage curves of the bacterial 16S rRNA gene amplicon paired-end sequencing data (coverage index at the OUT level). The rhizosphere soils were sampled from the control (CK) group, and the GXGL-4A (WT) and the Δ*amt*B (KO) treatment groups, respectively. Figure S12: Spearman correlation heatmap of the activities of soil enzymes and the dominant genera in the cucumber rhizosphere soils that treated with different NFB bacterial cells. S-UE: soil urease; S-CL: soil cellulase; S-ALPT: soil alkaline protease. *: *p* < 0.05; **: *p* < 0.01; ***: *p* < 0.001. Table S1: Quality control for the RNA-seq data of the *∆amt*B mutant and the wild type strain GXGL-4A. Table S2: Mapping ratio of the clean reads to the unigenes in the genome of *K*. *radicincitans* GXGL-4A.
