# Supplementary material for: Nitrogen-Fixing Bacterium GXGL-4A Promotes the Growth of Cucumber Plant Under Nitrogen Stress by Altering the Rhizosphere Microbial Structure
Source: Microorganisms. 2025 Aug 5;13(8):1824. doi: 10.3390/microorganisms13081824 (PMC12388777; doi:10.3390/microorganisms13081824)
Supplement: Supplementary file 1 [file microorganisms-13-01824-s001.zip › Table S1 Quality control for RNA-seq data.pdf]

Table S1 Quality control for the RNA-seq data of the Δ*amt*B mutant and the wild type strain GXGL-4A

Sample name	Raw reads	Raw bases (bp)	Raw Q20 (%)	Raw Q30 (%)	Clean reads	Clean bases (bp)	Clean Q20 (%)	Clean Q30 (%)
KO_3	23823810	3597395310	96.59	92.57	23035630	3118700374	98.17	94.76
KO_2	25832590	3900721090	97.09	93.5	25145718	3472064871	98.38	95.27
KO_1	23248504	3510524104	96.8	93.14	22550792	3072582381	98.33	95.2
WT_3	28052334	4235902434	96.36	92.52	27286476	3533125673	98.32	95.22
WT_2	27968910	4223305410	96.33	92.41	27055950	3532254980	98.3	95.1
WT_1	27637222	4173220522	96.35	92.46	26633390	3429602313	98.35	95.28
