# Supplementary material for: Nitrogen-Fixing Bacterium GXGL-4A Promotes the Growth of Cucumber Plant Under Nitrogen Stress by Altering the Rhizosphere Microbial Structure
Source: Microorganisms. 2025 Aug 5;13(8):1824. doi: 10.3390/microorganisms13081824 (PMC12388777; doi:10.3390/microorganisms13081824)
Supplement: Supplementary file 1 [file microorganisms-13-01824-s001.zip › Table S2.pdf]

Table S2 Mapping ratio of the clean reads to the unigenes in the genome of *K. radicincitans* GXGL-4A

Sample Name	Total Reads	Genome Mapped Reads	Genome Mapped Ratio (%)	Unmapped Reads	Unmapped Reads Ratio (%)	Uniq Mapped Reads	Uniq Mapped Reads Ratio (%)
KO_1	22550792	22248201	98.66	302591	1.34	22096772	97.99
KO_2	25145718	24905684	99.05	240034	0.95	24730475	98.35
KO_3	23035630	22773121	98.86	262509	1.14	22605964	98.13
WT_1	26633390	26419017	99.2	214373	0.8	26210688	98.41
WT_2	27055950	26823958	99.14	231992	0.86	26608477	98.35
WT_3	27286476	27027717	99.05	258759	0.95	26745001	98.02
