# Supplementary figures and images for: Nitrogen-Fixing Bacterium GXGL-4A Promotes the Growth of Cucumber Plant Under Nitrogen Stress by Altering the Rhizosphere Microbial Structure
Source: Microorganisms. 2025 Aug 5;13(8):1824. doi: 10.3390/microorganisms13081824 (PMC12388777; doi:10.3390/microorganisms13081824)

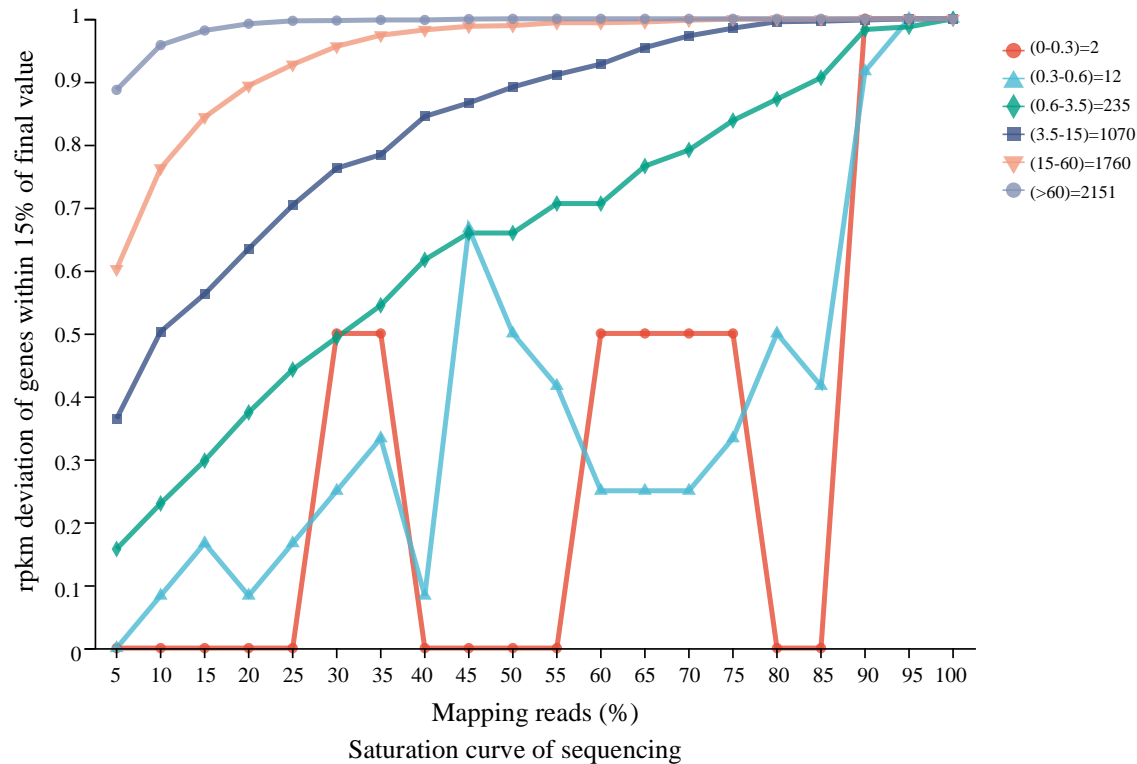

Supplement: Supplementary file 1 [file microorganisms-13-01824-s001.zip › Figure S1.pdf]

## Shannon curves

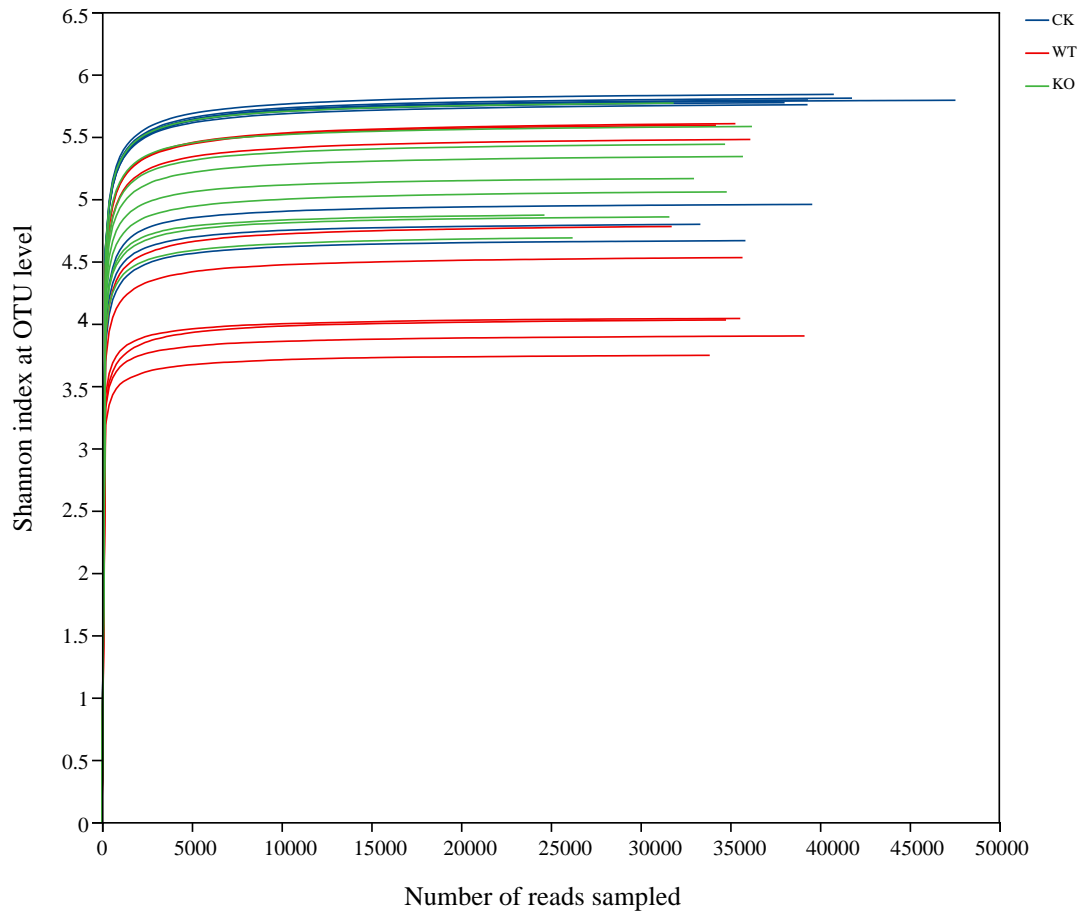

Supplement: Supplementary file 1 [file microorganisms-13-01824-s001.zip › Figure S10.pdf]

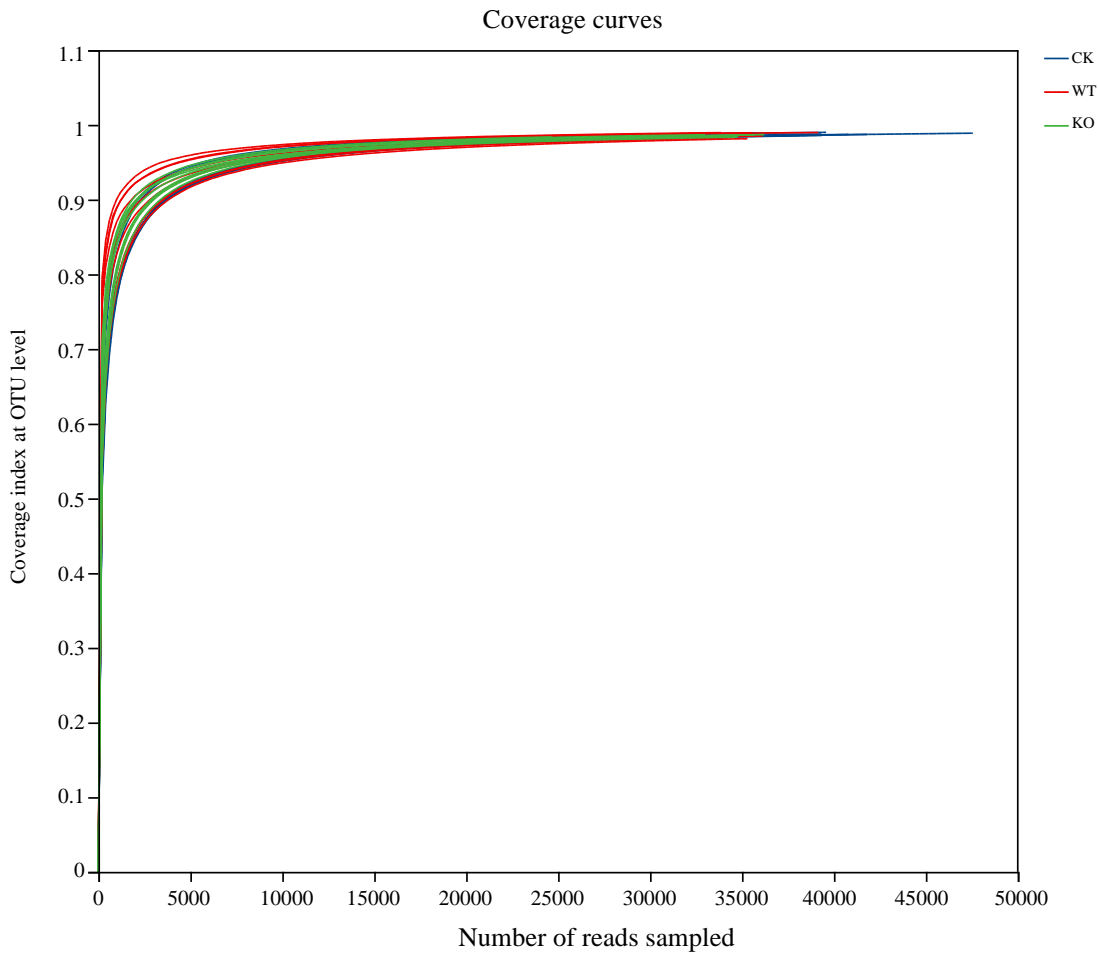

Supplement: Supplementary file 1 [file microorganisms-13-01824-s001.zip › Figure S11.pdf]

Spearman Correlation Heatmap

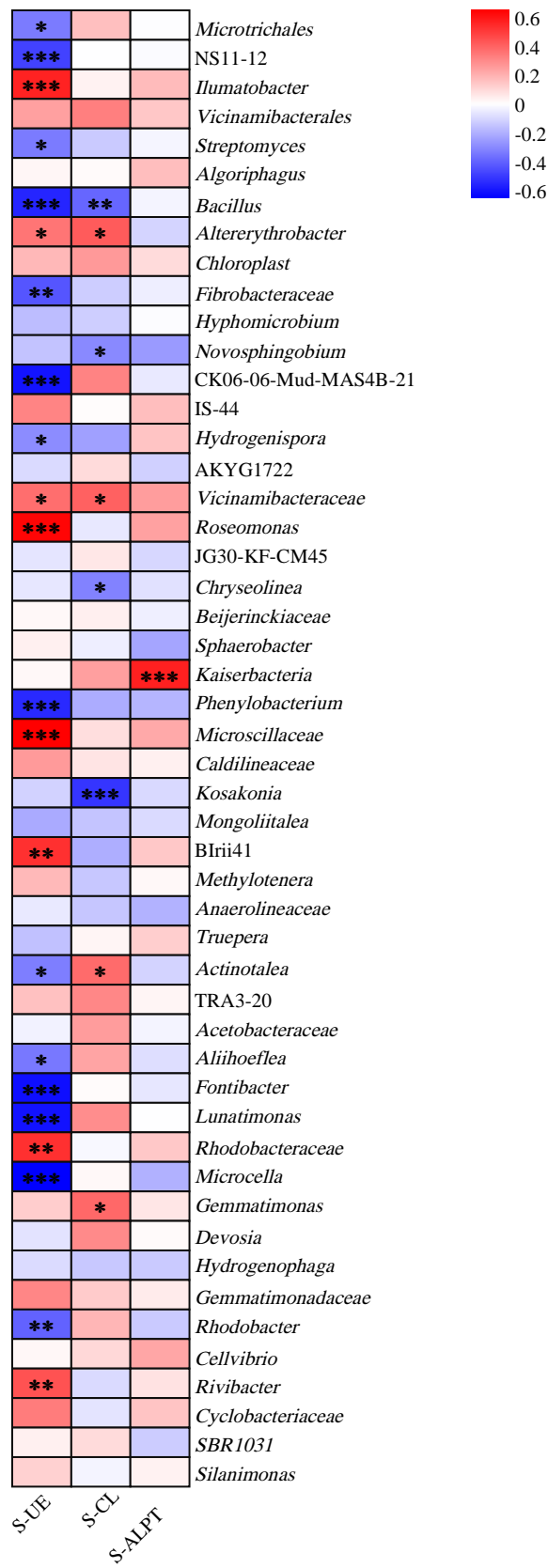

Supplement: Supplementary file 1 [file microorganisms-13-01824-s001.zip › Figure S12.pdf]

S2

## Coverage of sequencing

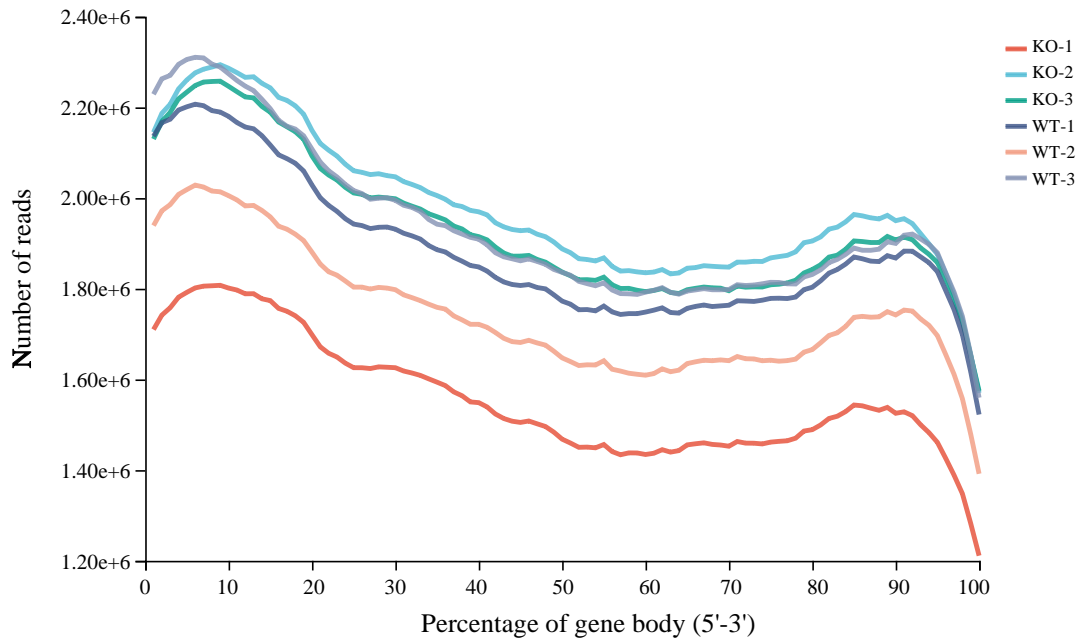

Supplement: Supplementary file 1 [file microorganisms-13-01824-s001.zip › Figure S2.pdf]

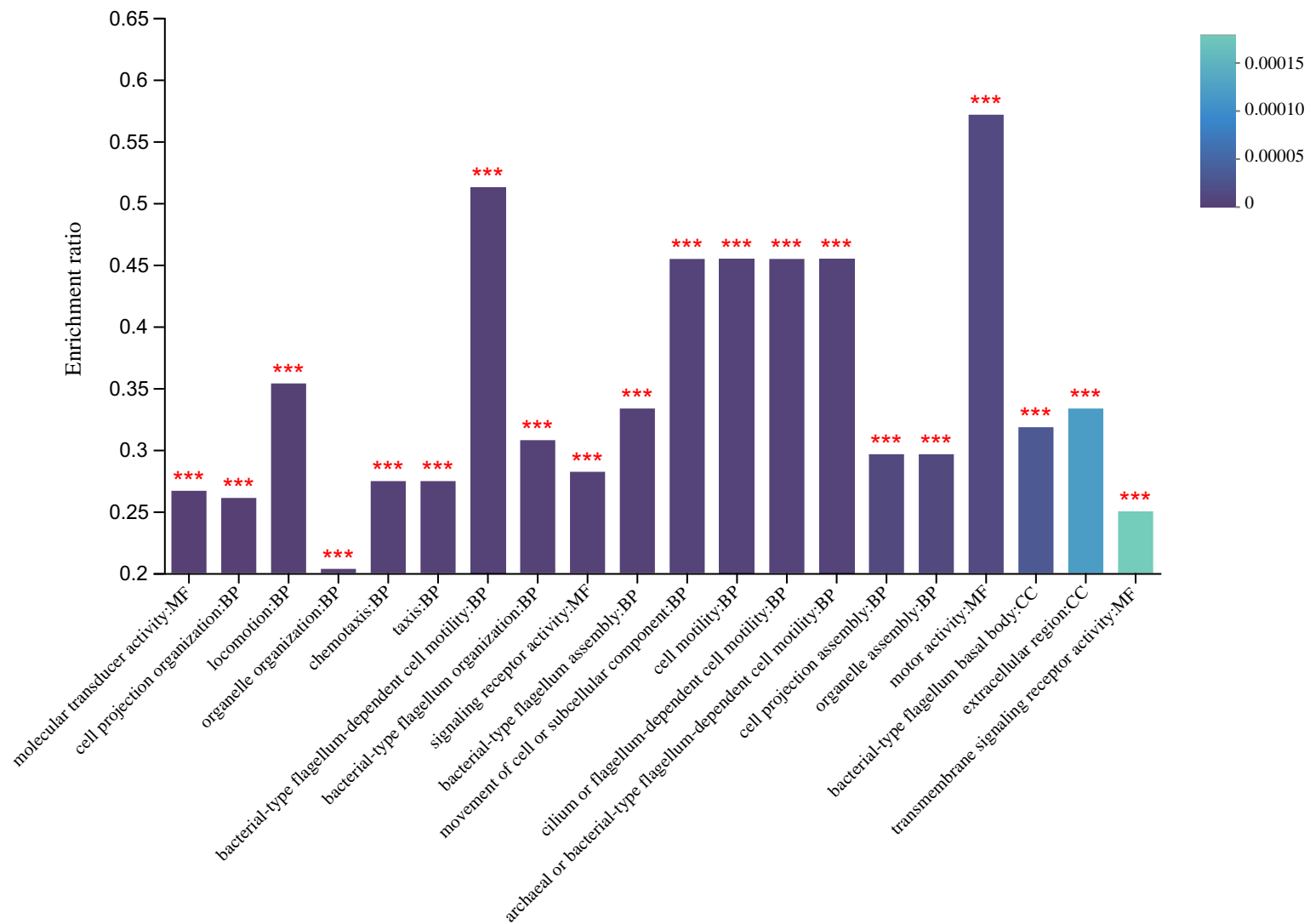

Supplement: Supplementary file 1 [file microorganisms-13-01824-s001.zip › Figure S3.pdf]

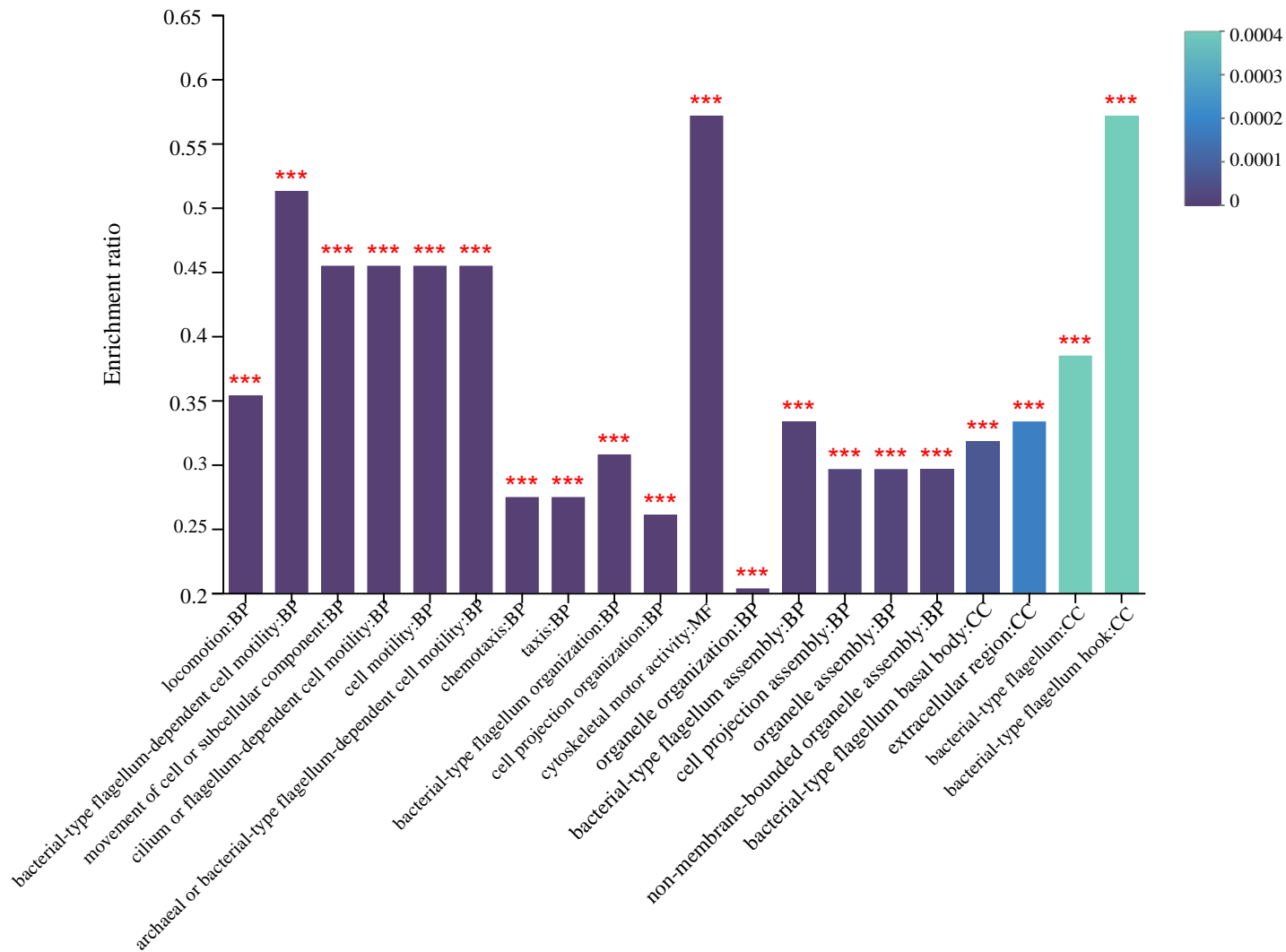

Supplement: Supplementary file 1 [file microorganisms-13-01824-s001.zip › Figure S4.pdf]

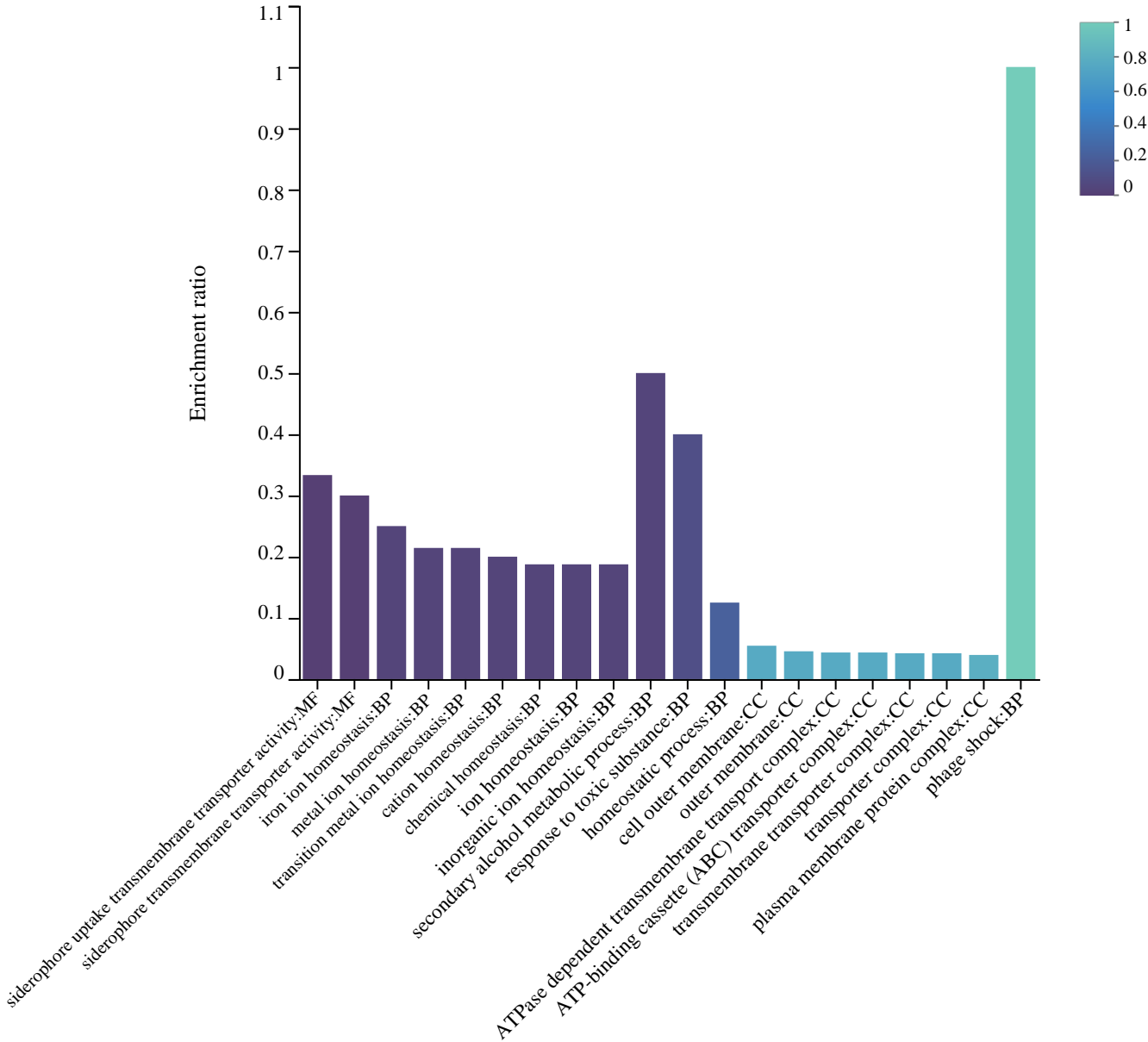

Supplement: Supplementary file 1 [file microorganisms-13-01824-s001.zip › Figure S5.pdf]

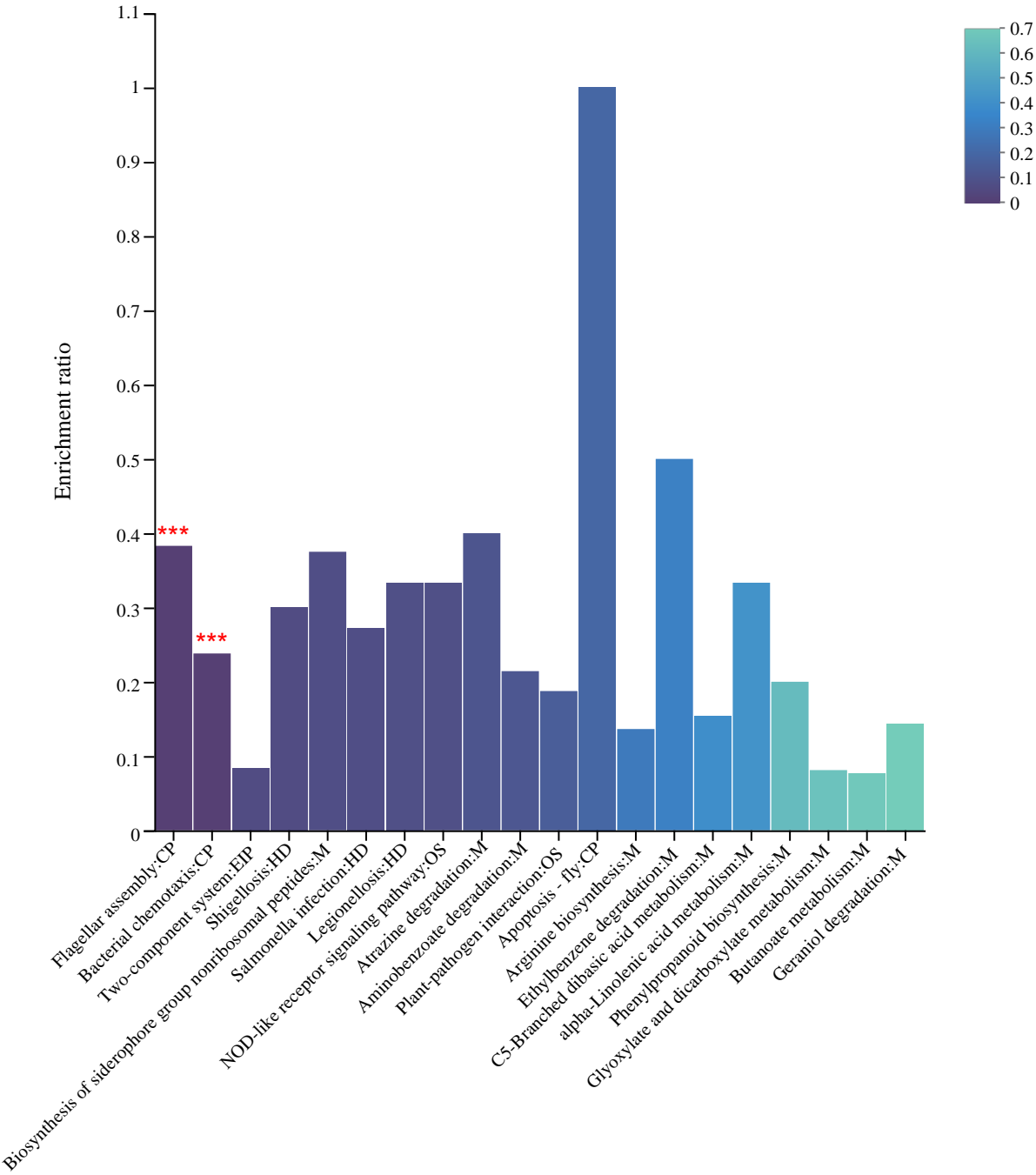

Supplement: Supplementary file 1 [file microorganisms-13-01824-s001.zip › Figure S6.pdf]

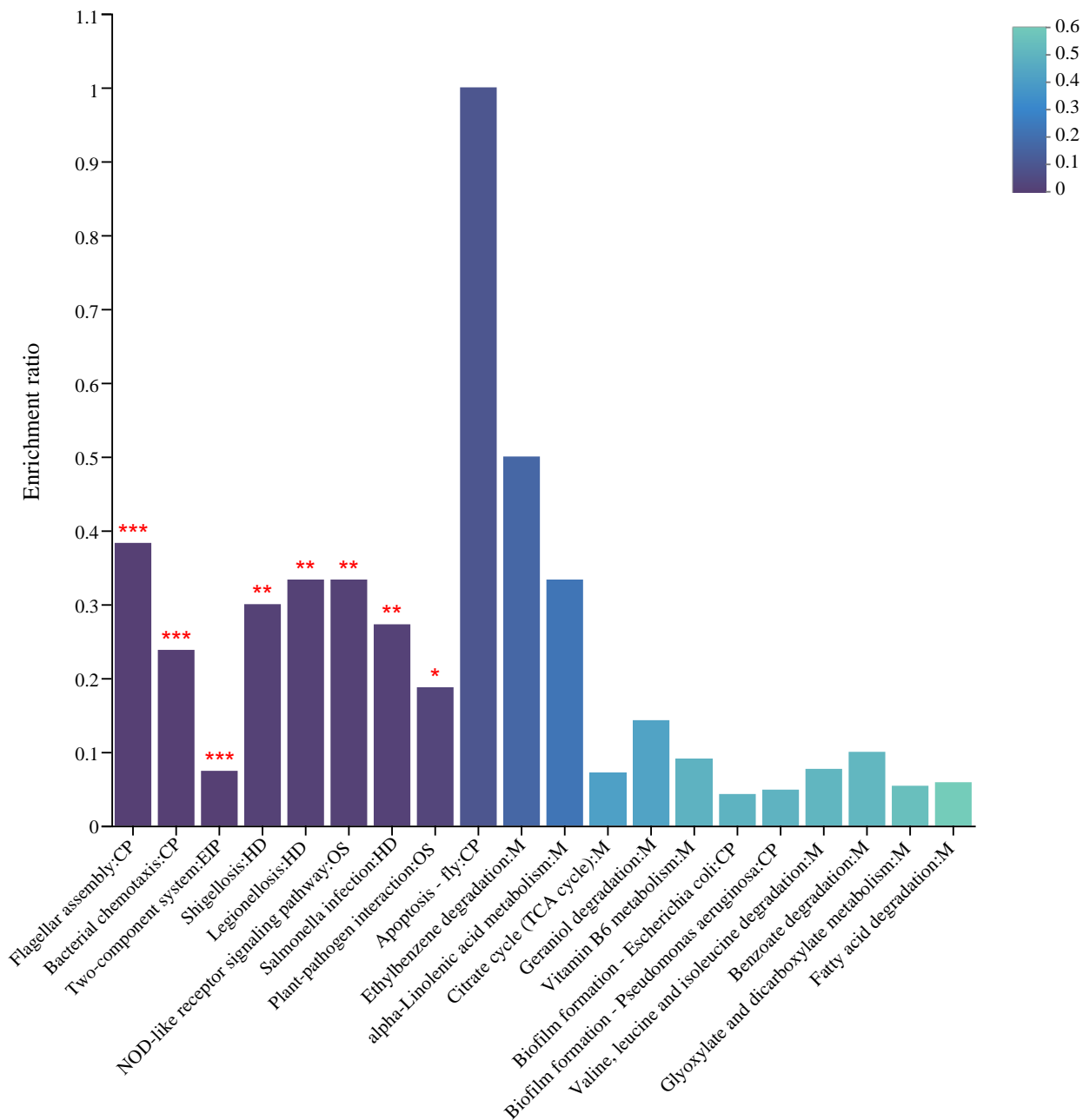

Supplement: Supplementary file 1 [file microorganisms-13-01824-s001.zip › Figure S7.pdf]

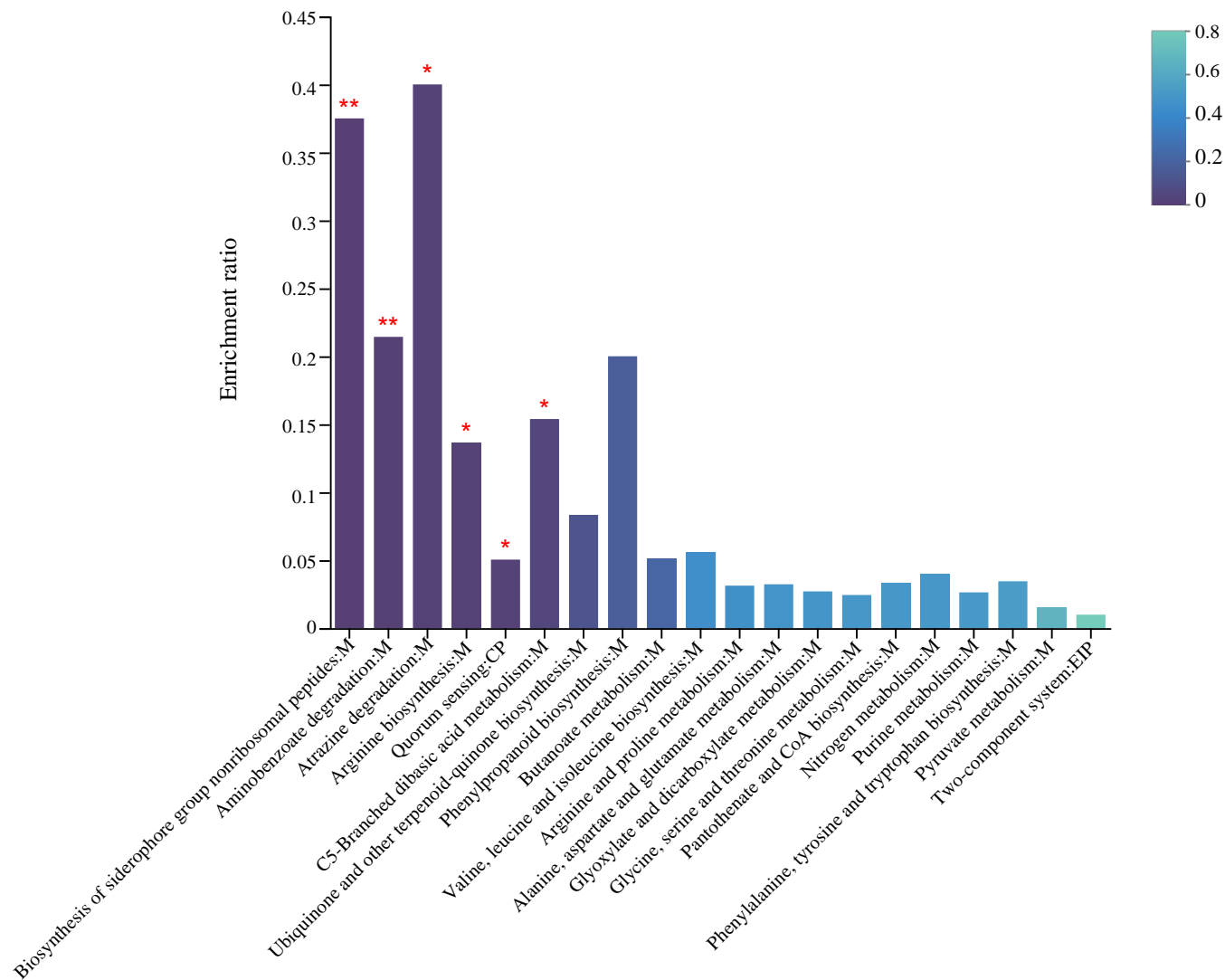

Supplement: Supplementary file 1 [file microorganisms-13-01824-s001.zip › Figure S8.pdf]

S9

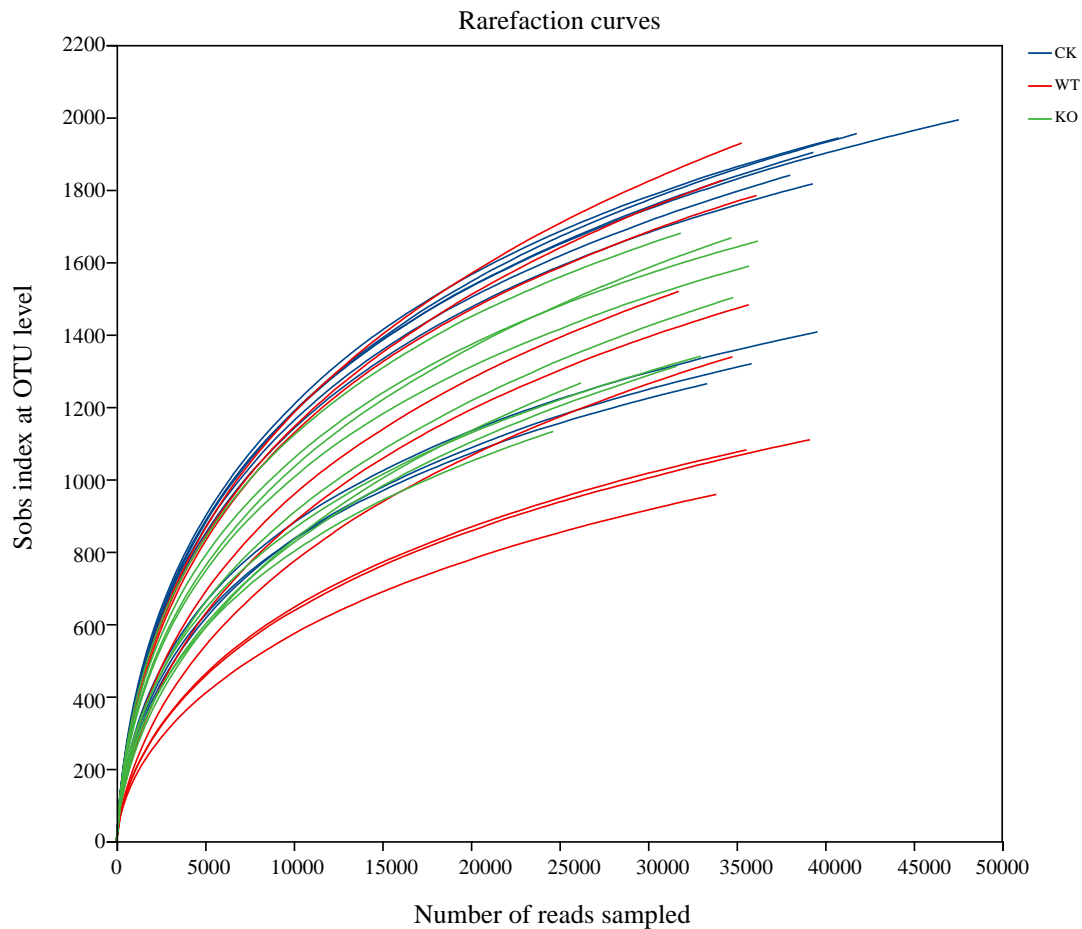

Supplement: Supplementary file 1 [file microorganisms-13-01824-s001.zip › Figure S9.pdf]
